# Heat shock proteins in osteoarthritis: molecular mechanisms, pathogenic roles, and therapeutic opportunities

**DOI:** 10.3389/fimmu.2025.1688250

**Published:** 2025-12-15

**Authors:** Bin Zhao, Zhijun Deng, Zhijun Yang, Fengyun Yang, Wenlong Yang

**Affiliations:** 1 Department of Orthopedics, Hospital Affiliated to Jiangxi University of Chinese Medicine, Nanchang, China; 2 Department of Orthopedics, Lishui Central Hospital, Lishui, China; 3 Graduate School, Jiangxi University of Chinese Medicine, Nanchang, China

**Keywords:** osteoarthritis, heat shock proteins, endoplasmic reticulum stress, chondrocytes, inflammation, GRP78, HSP70, therapeutic targets

## Abstract

Osteoarthritis (OA) is the most prevalent degenerative joint disease, characterized by cartilage degradation, chondrocyte apoptosis, synovial inflammation, and subchondral bone remodeling. Accumulating evidence highlights the central role of heat shock proteins (HSPs) in OA pathogenesis and progression. HSPs function as molecular chaperones that maintain proteostasis by facilitating protein folding, preventing aggregation, and modulating stress responses. Dysregulated expression of HSP27, HSP40, HSP60, HSP70, HSP90, and GRP78 contributes to inflammation, extracellular matrix breakdown, and chondrocyte apoptosis, but also provides cytoprotective effects under certain conditions. This duality positions HSPs as both biomarkers of disease activity and promising therapeutic targets. Here, we comprehensively review the roles of HSPs in regulating apoptosis, autophagy, and inflammatory signaling in OA. We further discuss emerging therapeutic strategies that modulate HSP expression or activity, including synthetic drugs, natural products, nanomedicine, stem cell therapy, physical modalities (heat, ultrasound, phototherapy, microwaves), and biological agents such as monoclonal antibodies. By integrating mechanistic insights and translational advances, this review underscores the potential of HSP-targeted therapies to preserve chondrocyte function, maintain extracellular matrix integrity, and slow OA progression, paving the way for novel disease-modifying interventions.

## 1 Introduction

Osteoarthritis (OA) is a common and progressively increasing musculoskeletal disease, affecting about 7.6% of the global population as of 2020, and is projected to rise by 60–100% by 2050 (1). It disproportionately impacts women and primarily affects the knee, contributing significantly to disability worldwide, especially after the age of 70 (2). Key risk factors include age, obesity, and joint trauma, with a notable number of cases developing before the age of 55 (3). OA reduces quality of life due to persistent joint pain and diminished physical function and imposes considerable economic burdens through lost productivity and healthcare costs (4). Abnormal mechanical loading of articular cartilage triggers a network of molecular events that drive OA progression. Excessive load activates pro-inflammatory cytokines such as interleukin-1β (IL−1β) and tumor necrosis factor−α (TNF−α), which then stimulate downstream pathways like mitogen−activated protein kinases (MAPKs) and nuclear factor kappa-B (NF−κB), resulting in increased expression of matrix-degrading enzymes (e.g., MMPs and ADAMTS) and subsequent extracellular matrix (ECM) degradation (5). Endoplasmic reticulum stress (ERS) occurs when the endoplasmic reticulum (ER) becomes overwhelmed by the accumulation of unfolded or misfolded proteins, triggering a cellular stress response known as the unfolded protein response (UPR) (6). In OA, chondrocytes, the only cells in cartilage, experience ER stress due to increased demands for synthesizing extracellular matrix proteins and the presence of various stressors (e.g., inflammation, oxidative stress, mechanical overload). This stress can lead to chondrocyte dysfunction or death via apoptosis, contributing significantly to cartilage degradation and disease progression (7). Consequently, targeting ER stress components holds promise for novel OA treatments that restore cellular homeostasis, preserve chondrocyte viability, and ultimately slow or reverse cartilage deterioration (8). Heat shock proteins (HSPs) are central to the cellular proteostasis network, facilitating proper protein folding and degradation of misfolded proteins. Under ER stress, their chaperone activity cooperates with ER-resident proteins such as HSP70 to restore folding homeostasis and alleviate stress-induced damage (9, 10). Small HSPs such as HSP27 form dynamic oligomers that bind unfolding proteins in an ATP-independent manner, acting as “holdases” that prevent aggregation and subsequently hand off clients to ATP-dependent chaperones (11). HSP40 (DNAJ) proteins function mainly as co-chaperones: they recognize misfolded substrates and stimulate the ATPase activity of HSP70, thereby controlling both client capture and release (12). HSP60, primarily localized in mitochondria, assembles into barrel-shaped oligomers that encapsulate client proteins within a protected folding chamber (13). HSP70 family members are modular chaperones with an N-terminal nucleotide-binding domain and a C-terminal substrate-binding domain linked by a flexible hinge; ATP binding and hydrolysis drive conformational changes that alternately clamp and release client proteins, enabling iterative folding cycles and directing terminally misfolded proteins toward the ubiquitin–proteasome system or autophagy (14–16). HSP90 operates as a dimer with a conserved N-terminal ATP-binding domain, a middle client-binding domain, and a C-terminal dimerization domain, and cooperates with co-chaperones to stabilize kinases, transcription factors, and receptors that are central to stress and inflammatory signaling (17). Alterations in HSPs such as Hsp27, Hsp60, Hsp70, and Hsp90 play complex and dual roles in arthritis. They can drive inflammation by stimulating proinflammatory cytokines and activating immune cells, thereby exacerbating joint damage. Simultaneously, these proteins possess anti-inflammatory and cytoprotective properties by promoting regulatory T cell responses and limiting tissue degradation. This duality makes HSPs markers of disease activity and attractive targets for new therapeutic approaches that aim to balance their harmful and beneficial effects (18). In this review we provide a comprehensive, family-by-family overview of how HSP27, HSP40, HSP60, HSP70/GRP78, and HSP90 contribute to OA pathogenesis through context-dependent protective and deleterious effects on chondrocytes, synovium, and extracellular matrix homeostasis. Building on this mechanistic framework, the subsequent section focuses on available and emerging therapeutic interventions that target HSPs or HSP-regulated pathways, outlining pharmacological, biophysical, and lifestyle-based strategies and discussing their potential to restore proteostatic balance and modify disease progression.

## 2 Heat shock proteins overview

**Figure 1:**
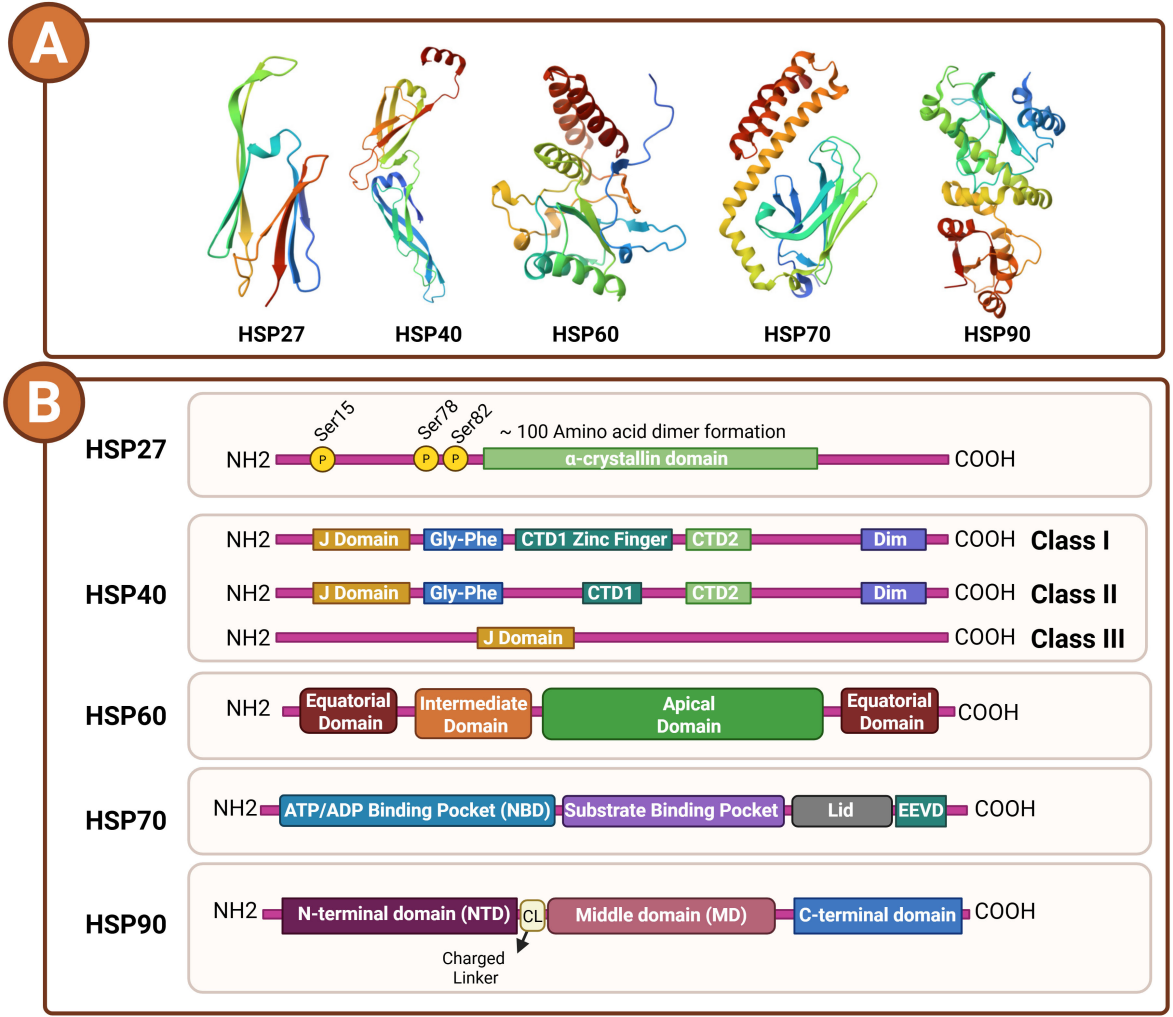
Structural and domain organization of major heat shock protein (HSP) families. **(A)** Representative three-dimensional ribbon structures of HSP27, HSP40, HSP60, HSP70, and HSP90, illustrating their distinct folding topologies and domain arrangements. Structures were retrieved from the Protein Data Bank (PDB) and visualized using color-coded secondary elements (α-helices, β-sheets, and loops). **(B)** Schematic representation of domain organization for each HSP family. HSP27 contains an N-terminal region with phosphorylation sites (Ser15, Ser78, Ser82) and a conserved α-crystallin domain responsible for dimerization. HSP40 proteins are categorized into three classes (I–III) based on the presence of the J-domain, glycine/phenylalanine-rich (Gly-Phe) regions, C-terminal domains (CTD1/CTD2), zinc-finger motifs, and dimerization modules. HSP60 consists of equatorial, intermediate, and apical domains forming a tetradecameric chaperonin structure. HSP70 features an N-terminal nucleotide-binding domain (NBD), substrate-binding domain (SBD), interdomain lid, and C-terminal EEVD motif for co-chaperone interactions. HSP90 comprises an N-terminal ATP-binding domain (NTD), a middle domain (MD) connected by a charged linker (CL), and a C-terminal dimerization domain (CTD).

Heat shock proteins are a universally conserved family of molecular chaperones present across all forms of life (19). They are pivotal in sustaining cellular protein homeostasis and safeguarding cells against diverse stressors, including elevated temperature, oxidative damage, and toxic insults (10, 20). Their functions encompass guiding the proper folding of nascent polypeptides, rescuing misfolded or aggregated proteins, facilitating intracellular protein transport, and modulating key processes such as apoptosis and immune regulation. Classification of HSPs is primarily based on their molecular mass, expressed in kilodaltons (kDa), a feature that not only denotes their size but also aligns with specific structural properties and specialized biological roles (10). Herein, we briefly review each HSP family according to their structural domain organization (**Figure 1**). Also, we classified the HSP family proteins with their specification and functions in **Table 1**.

**Table 1:** Summary of the structural class, molecular characteristics, primary functions, client proteins, pathway associations, and disease relevance of HSPs.

HSP name	Family/Class	Molecular weight	Main functions	Molecular targets	Pathway involvement	Ref.
HspB1 (Hsp27)	Small heat shock protein (sHsp)	~27 kDa (monomer)	Chaperone activity, prevention of protein aggregation, regulation of actin cytoskeleton, modulation of apoptosis, redox homeostasis	Denatured/misfolded proteins, actin filaments, pro-apoptotic factors (e.g., cytochrome c, caspase-3), signaling molecules (e.g., STAT3, Akt), transcription factors (e.g., NF-κB components)	MAPK-p38–MK2 phosphorylation pathway, Akt signaling, NF-κB activation, oxidative stress response, apoptosis regulation	(21)
Hsp40 (J-domain proteins, DnaJ homologs)	Hsp40 family, Classes I–III	~40 kDa (monomer)	Co-chaperone for Hsp70; stimulates Hsp70 ATPase activity; delivers unfolded or partially folded polypeptides to Hsp70; prevents protein aggregation; substrate scanning	Unfolded/misfolded polypeptides (hydrophobic residue–rich), Hsp70-ATP complex	Hsp70 chaperone cycle, protein folding, protein import into organelles (e.g., ER), degradation pathways (ERAD), disaggregation machinery	(22)
HSP60	Chaperonin (Group I)	~60 kDa (monomer)/~800 kDa (tetradecamer complex)	Protein folding and refolding, mitochondrial protein import, prevention of protein aggregation, assistance in assembly of multi-protein complexes, regulation of apoptosis and immune responses	Mitochondrial matrix proteins, nuclear-encoded mitochondrial enzymes, unfolded/misfolded proteins, apoptotic regulators (procaspase-3, Bax), TLR4 and TLR2 (extracellular HSP60)	Mitochondrial unfolded protein response (mtUPR), apoptosis signaling, innate immune signaling (TLR2/TLR4-NF-κB), stress response pathways	(13)
Hsp70 (HspA1A/HspA1B, inducible)	Cytosolic stress-inducible Hsp70 family	~70 kDa	Protein folding, prevention of aggregation, degradation of oxidized proteins, regulation of antioxidant enzymes, redox sensing via cysteine modifications	SOD2, Hsf1, Akt1, CHIP (STUB1), oxidized proteins	PI3K/Akt/Hsp70, JAK2/STAT3/Hsp70, Keap1/Nrf2/Hsf1/Hsp70	(23)
HSP 90 (HspA1A/HspA1B (Inducible), HspA8 (Hsc70), HspA9 (Grp75, mortalin), HspA5 (Grp78, BiP), HspH1 (Hsp105), PfHsp70-1, PfHsp70-2)	Cytosolic stress-inducible Hsp70, cytosolic constitutive Hsp70, mitochondrial Hsp70, ER Hsp70, Hsp110-like NEF, parasite cytosolic Hsp70, parasite ER Hsp70	~70–105 kDa	Protein folding, prevention of aggregation, degradation of oxidized proteins via CMA, antioxidant defense, redox regulation, mitochondrial protein import, ER protein quality control, unfolded protein response, NEF activity for Hsp70, oxidative stress tolerance in parasites	SOD2, Hsf1, Akt1, CHIP (STUB1), oxidized proteins, lysosomal CMA substrates, mitochondrial proteins, ER nascent/misfolded proteins, parasite oxidized proteins	PI3K/Akt/Hsp70, JAK2/STAT3/Hsp70, Keap1/Nrf2/Hsf1/Hsp70, UPR, ROS-mediated signaling	(24)

### 2.1 HSP27

HSP27, also known as HSPB1, is one of the small heat shock proteins (sHSPs) (25). It is a chaperone protein that is categorized according to its distinct domain organization, rather than ATPase activity (26). HSP27 does not contain an ATP-binding domain as seen in HSP70 or HSP90, but rather has a conserved α-crystallin domain (containing about 80–100 amino acids) in its center (27), and is associated with a variable N-terminal WD/EPF-rich domain and a disordered C-terminal tail (28). The N-terminal domain appears to be involved in the regulation of oligomerization and phosphorylation-dependent dynamic activity (29), and the α-crystallin domain is responsible for dimerization and substrate binding, which is the chaperone core (30). The IXI/V-rich C-terminal domain plays a role in stabilizing the large oligomeric assembly, as well as mediating client protein interaction (31). Phosphorylation at conserved serine residues (Ser15, Ser78, Ser82) results in dissociation of the large 200–700 kDa oligomers into active dimers or tetramers, with a functional effect on its chaperone and cytoprotective properties (32).

### 2.2 HSP40

HSP40 (DNAJ) proteins are the most diverse family of molecular cochaperones, and are characterized by a conserved ~70 amino acid J-domain required for activation of HSP70 ATPase activity and for the orderly transfer of substrates between the two chaperones during protein folding (33). HSP40s are further grouped into 3 major classes, on the basis of domain architecture. Class I HSP40s (e.g. E. coli DnaJ) are typically composed of 5 domains. These are the N-terminal J-domain which contains the signature HPD motif required for HSP70 interaction; the glycine/phenylalanine-rich (G/F) region which imparts flexibility to the protein; the C-terminal domain 1 (CTD1) which contains a zinc-finger–like region (ZFLR) and mediates client binding; the C-terminal domain 2 (CTD2) which stabilizes the fold of the protein; and a dimerization (D) domain which enables the formation of functional dimers. Class II HSP40s have a similar overall architecture to class I members but lack the ZFLR in CTD1. Members of Class III are the most divergent and retain only the J-domain. In some of these proteins, the J-domain is not at the N-terminus, but at a variable position in the sequence (22). The structural plasticity of HSP40s underlies the functional heterogeneity within this group. HSP40s can function as general folding assistants or specialized cochaperones that target specific sets of substrates and cellular pathways, along with HSP70 (34).

### 2.3 HSP60

The HSP60 family, also known as chaperonins, is classified on the basis of its conserved structural domain organization that underlies its unique folding mechanism (35). HSP60 forms a double-ring cylindrical complex, typically composed of 14 identical subunits arranged in two heptameric rings stacked back-to-back, creating a central cavity where protein folding occurs (36). Each subunit contains three structurally distinct domains: an equatorial domain, which harbors the ATP-binding site and mediates inter-ring contacts (37); an intermediate domain, which acts as a flexible hinge transmitting conformational changes (38); and an apical domain, which binds non-native polypeptides and interacts with the co-chaperonin HSP10 (GroES) that functions as a lid (39). This highly conserved architecture is shared across species, from bacterial GroEL/GroES systems to eukaryotic mitochondrial HSP60/HSP10 complexes (40), and defines the Group I chaperonins, distinguishing them from the Group II chaperonins (CCT/TRiC) that lack a separate co-chaperonin lid (13).

### 2.4 HSP70

The Hsp70 family is classified on the basis of its conserved structural domain organization, which defines both its mechanistic cycle and functional versatility (41). Each Hsp70 molecule consists of three principal domains: an N-terminal nucleotide-binding domain (NBD) that hydrolyzes ATP and drives allosteric conformational changes (42); a substrate-binding domain (SBD) composed of a β-sandwich subdomain (SBDβ) forming the peptide-binding groove and an α-helical lid (SBDα) that stabilizes bound substrates (43); and a C-terminal tail that, in cytosolic isoforms, ends with an EEVD motif acting as a docking site for co-chaperones and downstream protein quality control factors (44). This conserved domain framework enables the ATP-driven allosteric cycle characteristic of the Hsp70 chaperone system, coordinating with J-domain proteins (JDPs) for substrate capture and nucleotide-exchange factors (NEFs) for substrate release, thereby maintaining proteostasis across all cellular compartments (45). Glucose-Regulated Protein 78 (GRP78), also known as Binding immunoglobulin Protein (BiP), is a member of the HSP70 family that serves as the principal chaperone in the ER, orchestrating the UPR under stress conditions (46). Structurally, GRP78 shares the conserved domain organization characteristic of the HSP70 family, comprising two major domains including NBD and SBD (47).

### 2.5 HSP90

HSP90s are also classified according to their subcellular localization and their conserved domain architectures (9). HSP90s have a domain structure that is conserved in all of the different isoforms, and all three domains are essential for function (48). They all consist of a large N-terminal domain (NTD), a middle domain (MD) and a smaller C-terminal domain (CTD) (49). The NTD binds and hydrolyzes ATP, the MD is involved in client interaction and regulation of ATPase activity and the CTD is involved in dimerization (50). Between the NTD and MD is a charged linker region that allows for flexibility between the two domains and also regulates interactions between the domains (51). The mammalian cytosolic HSP90 proteins are inducible (HSP90α) and constitutive isoforms (HSP90β) (52), while HSP90 chaperones that reside in the endoplasmic reticulum (GRP94/GP96) and mitochondria (TRAP1) are also present in humans (53).

## 3 The role of HSPs in osteoarthritis pathogenesis

### 3.1 HSP27

HSP27 is a small heat shock protein that plays a crucial role in cellular protection and homeostasis (54). During fetal development and early childhood, HSP27 is highly expressed in the chondrocytes of the endplate cartilage, particularly in zones associated with chondrocyte maturation, suggesting a role in supporting metabolic and morphologic changes during tissue growth and differentiation (55). The cellular functions of HSP27 in OA are outlined below:

#### 3.1.1 Autophagy regulation

In OA, HSP27 contributes to delaying disease progression primarily by promoting autophagy, an essential process that helps cells remove damaged components and maintain metabolic balance. TSP-1, a protein shown to be protective in OA, upregulates HSP27 expression, which in turn enhances autophagy in chondrocytes, the primary cells in cartilage tissue. This autophagy activation mitigates cartilage degradation by preserving chondrocyte function, reducing inflammation, and preventing apoptosis. Therefore, HSP27 helps maintain cartilage integrity and slows OA progression by supporting cellular survival mechanisms and sustaining extracellular matrix (ECM) homeostasis (56).

#### 3.1.2 Chondrocyte metabolism regulation

HSP27 is crucial in preserving chondrocyte homeostasis by modulating the cellular response to inflammatory stimuli and regulating key aspects of chondrocyte metabolism. This heat shock protein is significantly downregulated in OA chondrocytes at protein and mRNA levels, particularly in response to pro-inflammatory cytokines like IL-1β and TNF-α. Functionally, HSP27 influences the expression of IL-6, a key inflammatory mediator, by stabilizing its mRNA, as shown through RNA interference experiments. Additionally, its expression increases during chondrocyte dedifferentiation in monolayer culture, indicating a role in phenotype regulation. However, this is not associated with changes in matrix protein genes such as collagen type II or aggrecan (57).

#### 3.1.3 Inflammation regulation

**Figure 2:**
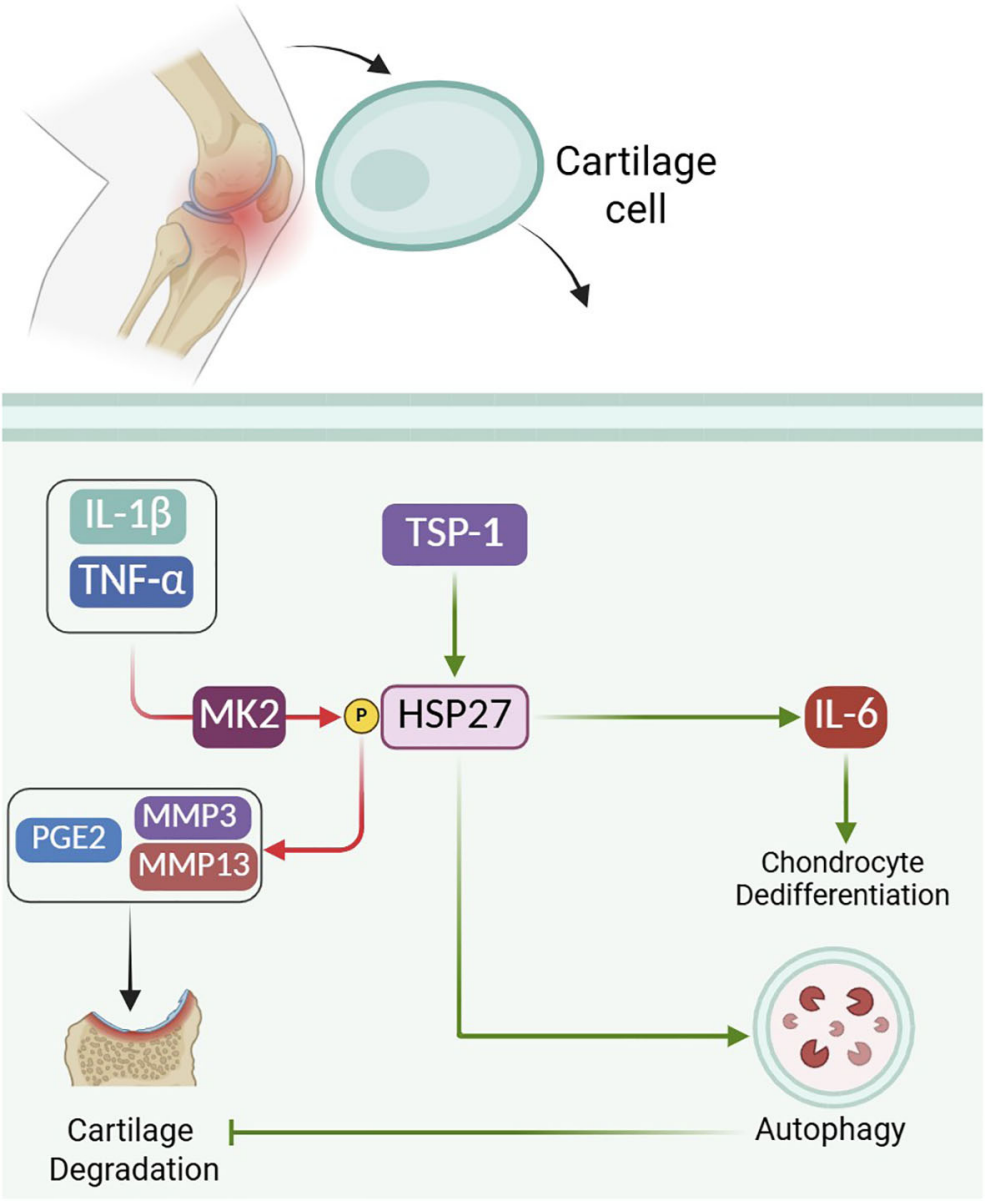
Role of HSP27 in osteoarthritis pathogenesis and chondrocyte homeostasis. Pro-inflammatory cytokines (IL-1β, TNF-α) activate the MK2 signaling pathway, leading to phosphorylation of HSP27. Phosphorylated HSP27 promotes PGE2 release and upregulates matrix metalloproteinases (MMP3, MMP13), contributing to cartilage degradation. Conversely, TSP-1 upregulates HSP27 expression, which enhances autophagy in chondrocytes and preserves cartilage integrity. HSP27 also regulates IL-6 expression and influences chondrocyte dedifferentiation. Together, these mechanisms highlight HSP27 as a key modulator of inflammation, catabolism, and cellular survival in osteoarthritis. Green lines indicate protective effects, while red lines represent harmful effects.

HSP27, as a downstream substrate of MK2, has a crucial role in the inflammatory cascade of OA by modulating key signaling pathways involved in disease pathology. Upon activating pro-inflammatory cytokines such as TNF-α and IL-1β, MK2 phosphorylates HSP27, a marker of MK2 pathway activation (58). This phosphorylation event is critical in mediating the production of prostaglandin E2 (PGE2), a pro-algesic mediator, as well as in regulating the expression of catabolic proteases like matrix metalloproteinases (MMP3 and MMP13) that contribute to cartilage degradation. The inhibition of HSP27 phosphorylation, either through dominant negative MK2 expression or MK2 siRNA knockdown, significantly reduces cytokine-induced PGE2 release and MMP expression, indicating that phosphorylated HSP27 acts as a pivotal effector in the p38-MK2 signaling axis contributing to inflammation and cartilage catabolism in OA (**Figure 2**) (59). Taken together, these findings indicate that HSP27 exerts an overall chondroprotective effect in OA by promoting autophagy and supporting chondrocyte homeostasis, but its phosphorylated form within the p38–MK2 pathway can also amplify inflammatory and catabolic responses, underscoring a context- and signaling-dependent net impact on OA progression.

### 3.2 HSP40

The Hsp40 protein family, also known as DNAJ proteins, is an essential molecular chaperones that regulate protein folding and quality control within cells, particularly in the ER (60). Among them, DNAJB12 plays a key role by guiding Hsp70 chaperones to the ER surface and participating in the degradation of misfolded proteins through ER-associated autophagy (ERAA), helping maintain intracellular protein homeostasis (61). In OA, dysfunction in protein quality control and autophagy pathways is increasingly recognized as contributing to disease progression. DNAJB12 is identified as a potential risk factor for knee OA. Thus, targeting Hsp40 family members, such as DNAJB12, could offer new therapeutic strategies for OA by restoring proper protein handling and reducing ER stress, thus potentially slowing or altering disease progression (62). HSP40 family members remain largely understudied in OA, and their precise roles in disease pathogenesis require further experimental investigation.

### 3.3 HSP60

HSP60 is a highly conserved molecular chaperone involved in protein folding and cellular stress responses (63). Hsp60, a mitochondrial chaperone protein, is crucial in maintaining mitochondrial function and protecting cells from oxidative stress-induced damage (64).

#### 3.3.1 HSP60 levels in OA

In OA, HSP60 expression and its immunogenic role appear to be limited compared with autoimmune conditions. Unlike rheumatoid arthritis (RA), where anti-HSP60 autoantibodies are abundant and contribute to disease pathogenesis (65), OA patients generally show low or undetectable levels of HSP60 and its corresponding antibodies. This suggests that HSP60-related autoimmunity is not a prominent mechanism in OA, and HSP60 may instead play a role in cellular stress responses and chondrocyte homeostasis rather than in immune-mediated joint damage (66). In patients with OA, the circulating level of HSP60 is significantly decreased compared to healthy controls. This reduction is observed in OA patients without a history of COVID-19 and those who recovered from SARS-CoV-2 infection, suggesting that the decrease in HSP60 may be a feature of joint pathology rather than a direct consequence of viral infection (67).

#### 3.3.2 Regulating inflammation

**Figure 3:**
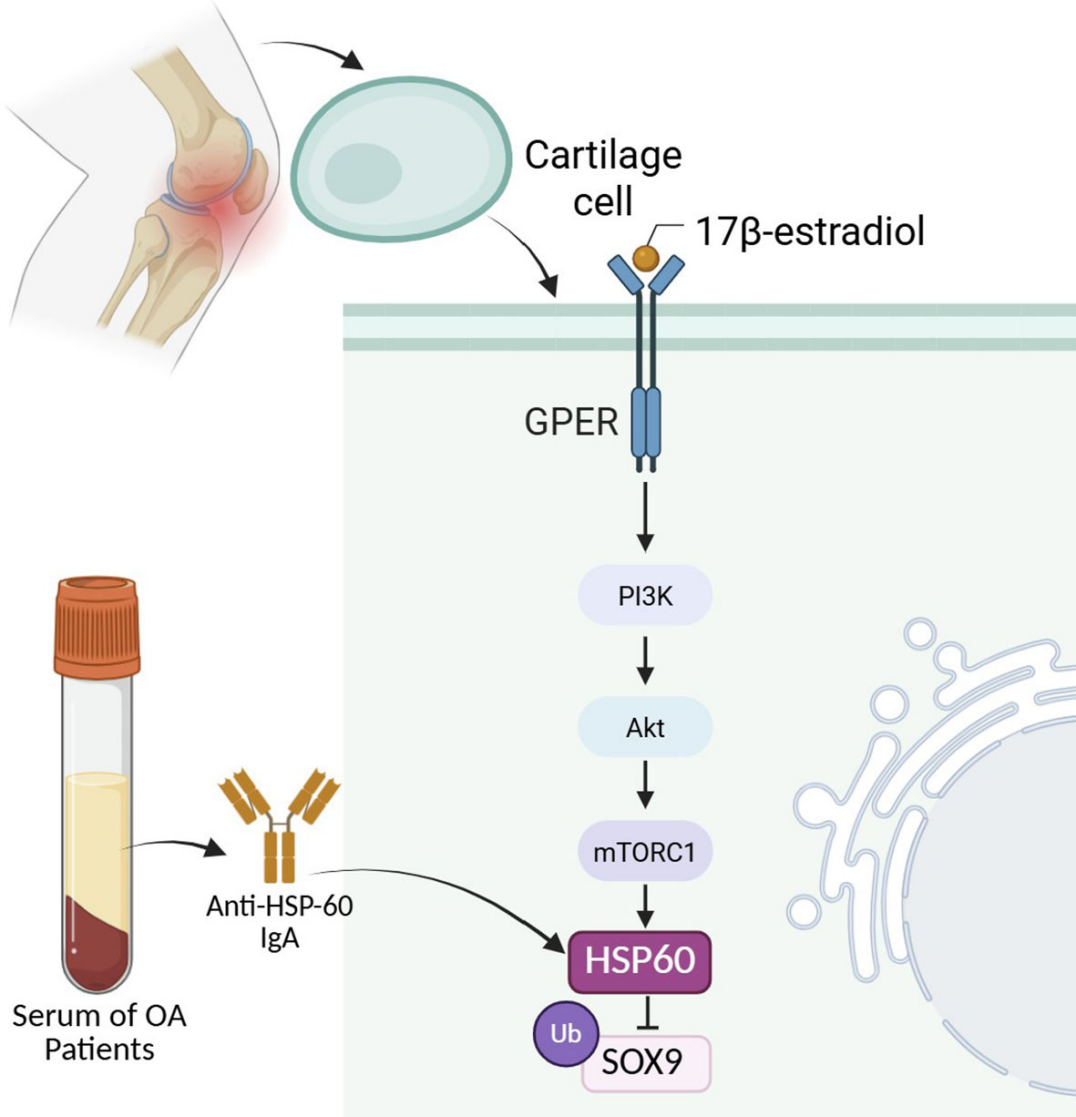
The role of HSP60 in chondrocyte homeostasis and osteoarthritis pathogenesis. HSP60 expression in chondrocytes is upregulated by 17β-estradiol through GPER-mediated activation of the PI3K/Akt/mTORC1 signaling pathway. Elevated HSP60 stabilizes SOX9 by reducing its ubiquitination, thereby promoting cartilage matrix protein expression and preserving chondrocyte phenotype. In OA, serum from patients contains elevated anti-HSP60 IgA autoantibodies, indicating immune recognition of HSP60. Reduced HSP60 levels in OA cartilage contribute to impaired mitochondrial function, diminished stress responses, and decreased anti-inflammatory capacity, thereby accelerating cartilage degeneration and joint pathology.

HSP60 is known for its role in protein homeostasis and modulation of immune and inflammatory responses (68). Its lowered plasma concentration in OA could reflect impaired cellular stress responses and diminished anti-inflammatory capacity, potentially contributing to disease progression (67). 17β-estradiol increases the expression of Hsp60 in ATDC5 chondrocytes, a mouse chondrogenic cell line derived from teratocarcinoma cells (69). The upregulation of Hsp60 following 17β-estradiol treatment was significantly reduced when cells were co-treated with the GPER/GPR30 antagonist G15 or the PI3K inhibitor LY294002, indicating that this effect is mediated through the GPER/GPR30–PI3K/Akt–mTOR signaling pathway (69). HSP60 plays a protective role in the pathogenesis of OA by preserving cartilage integrity and mitigating synovial inflammation (**Figure 3**) (70). This molecular chaperone is deficient in damaged cartilage of OA patients, correlating with reduced levels of the chondrogenic transcription factor SOX9. Restoring or overexpressing HSP60 in chondrocytes enhances mitochondrial function, promotes cartilage matrix protein expression (such as collagen II and aggrecan), and suppresses inflammatory mediators like IL-1β and VEGF. Ubiquitination is a process by which a small regulatory protein called ubiquitin is attached to a target protein, marking it for degradation by the proteasome or altering its cellular function or location (71). HSP60 also reduces SOX9 ubiquitination, stabilizing this key cartilage homeostasis regulator. Transgenic mice with elevated HSP60 levels exhibit thicker cartilage, lower osteophyte formation, and improved gait patterns following OA induction. Furthermore, intra-articular administration of HSP60 attenuates joint damage and inflammation, suggesting its therapeutic potential in slowing OA progression (72).

#### 3.3.3 Local immune response

Human hsp60 elicited an elevated IgA antibody response in patients with OA, although the IgG response did not differ significantly from that of standard controls. This suggests that while HSP60 may not be directly involved in the systemic immune pathogenesis of OA as intensely as in rheumatoid arthritis, it may still play a role in local or mucosal immune responses associated with OA. The increased anti-HSP60 IgA levels, without elevated total serum IgA, imply a targeted immune recognition of HSP60, potentially reflecting tissue stress or damage within osteoarthritic joints. IgG and IgA antibody titers against HSP60 in sera of rheumatoid arthritis and OA patients (**Figure 3**) (73).

### 3.4 HSP70

**Figure 4:**
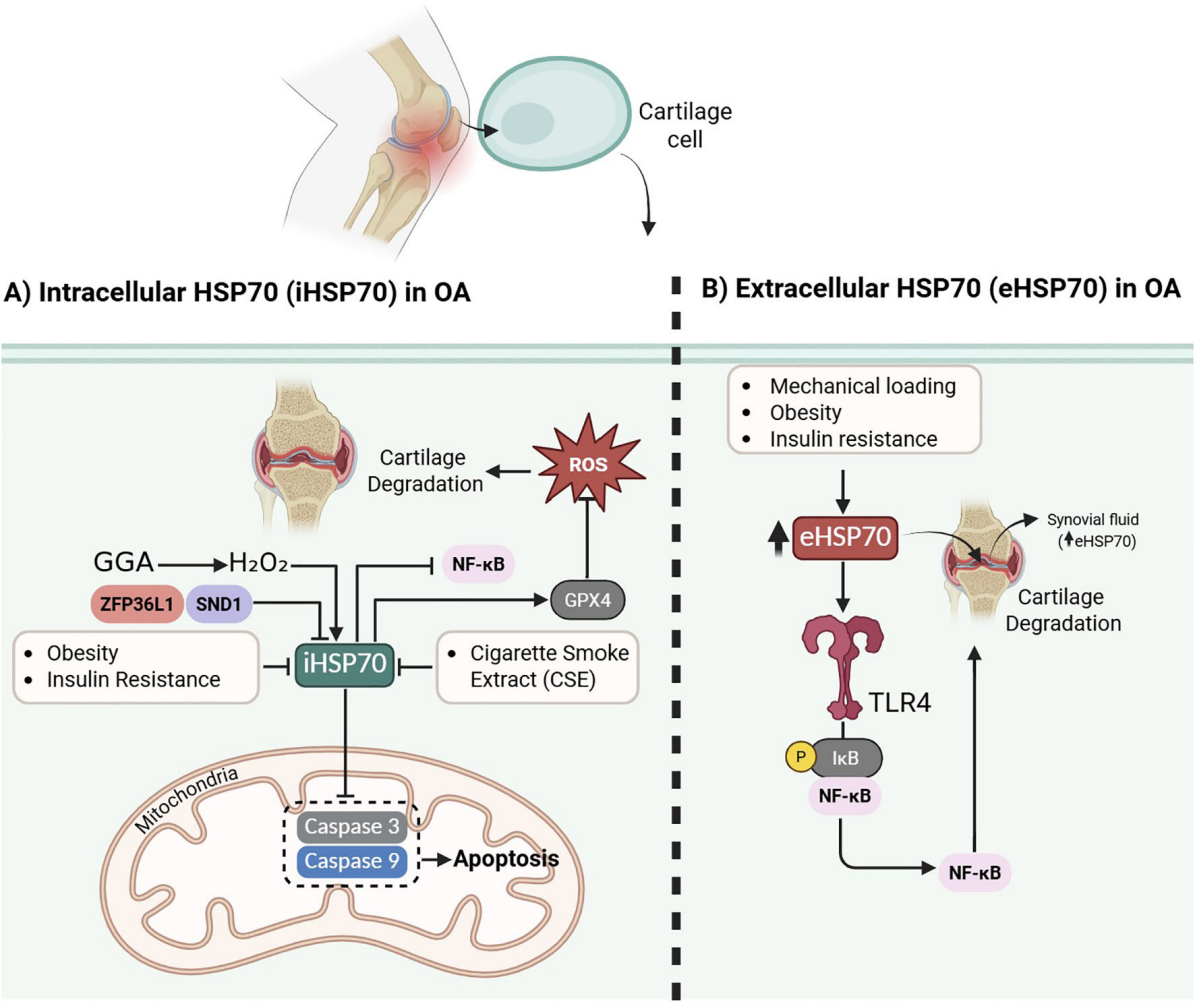
Dual role of HSP70 in osteoarthritis: protective intracellular effects versus pro-inflammatory extracellular signaling. Panel A: Intracellular HSP70 (iHSP70) exerts cytoprotective effects in chondrocytes by inhibiting caspase-3/9–mediated apoptosis, reducing oxidative stress, and maintaining mitochondrial and ECM integrity. iHSP70 expression is inhibited by obesity and insulin resistance, while it is activated by pharmacological activators such as GGA, but can be suppressed by CSE and regulatory proteins such as ZFP36L1 and SND1. Panel B: Conversely, extracellular HSP70 (eHSP70) is elevated in the synovial fluid of OA patients, especially in obesity, insulin resistance, and mechanical loading, and drives inflammation through TLR4–NF-κB signaling, contributing to synovitis and cartilage degradation. ROS and impaired GPX4 stability further amplify NF-κB activation, enhancing catabolism and OA progression. These contrasting functions highlight HSP70 as both a protective factor and a pathogenic mediator in OA, depending on its localization and regulation.

HSP70 is a molecular chaperone that maintains cellular protein homeostasis by assisting in proper protein folding, preventing aggregation, and facilitating protein repair or degradation (74). HSPs, particularly HSP70 (and its inducible form HSP72), play a pivotal role in the pathogenesis and potential management of OA (**Figure 4**) (75).

#### 3.4.1 Intracellular HSP70 vs. extracellular HSP70

HSP70 proteins are crucial regulators of cellular proteostasis and inflammation, with their actions differing based on location: intracellular HSP70 (iHSP70) exerts protective, anti-inflammatory, and anti-apoptotic effects, while extracellular HSP70 (eHSP70) promotes inflammation via Toll-like receptor (TLR) signaling (76). In OA, especially under conditions of obesity and insulin resistance, iHSP70 expression is suppressed due to impaired insulin signaling, while eHSP70 is elevated, contributing to synovitis, cartilage degradation, and disease progression (77). HSP70 expression correlates with OA severity and may serve as a biomarker (78). Therapeutic strategies that restore iHSP70 levels, such as exercise, heat therapy, and glutamine supplementation, could counteract synovial inflammation and cartilage loss, highlighting HSP70 as both a pathogenic factor and a potential therapeutic target in OA (75).

#### 3.4.2 Cartilage extracellular matrix regulation

HSP70 plays a crucial role in maintaining cartilage homeostasis under physiologically relevant thermomechanical conditions. Using 3D human chondrocyte, GelMA constructs, researchers found that combined hydrostatic pressure and temperature elevation (32.5–38.7°C) synergistically enhanced chondrogenic gene expression (SOX9, ACAN, COL2A) and mechanical strength, accompanied by a marked increase in HSP70 levels. Importantly, the data indicate that the thermal component is the dominant driver of HSP70 induction. When HSP70 was inhibited by quercetin, these beneficial effects were lost, thermal stimulation became detrimental, inducing catabolic and inflammatory markers (ADAMTS5, MMP3, COX2) and reducing construct stiffness. Proteomic analysis confirmed disrupted biosynthetic and cytoskeletal balance under HSP70 inhibition (79). In OA, HSP70 acts as a cellular protective mechanism against damage to cartilage and ECM components. HSP70 is upregulated in response to cigarette smoke extract (CSE) exposure, which induces oxidative stress (OS), inflammation, and ECM degradation through increased MMP9 and MMP13 expression and decreased antioxidant enzymes like catalase (CAT) and SOD1. The elevated expression of HSP70 in chondrocytes exposed to CSE suggests it stabilizes protein structure and protects cells from stress-induced apoptosis and ECM breakdown. This overexpression may represent an adaptive response to counteract damage, making HSP70 a potential biomarker of stress in OA and a possible therapeutic target to preserve cartilage integrity (80).

#### 3.4.3 Serum and synovial fluid levels of HSP70

Both circulating and synovial fluid levels of Hsp70 are significantly elevated in patients with primary knee OA compared to healthy controls, and these levels positively correlate with the radiographic severity of the disease as graded by the Kellgren and Lawrence (KL) classification. Notably, Hsp70 concentrations in synovial fluid were approximately three times higher than in plasma, and both increased proportionally with advancing KL grades (81).

#### 3.4.4 Inflammation regulation

HSP70, modulates various inflammatory signaling pathways in OA (18). Exposure to excessive hydrostatic pressure (e.g., 50 MPa) significantly upregulates HSP70 expression in chondrocytes, indicating its involvement as a stress response marker. This increase correlates with elevated NF-κB expression, suggesting that HSP70 may contribute to OA pathogenesis by activating NF-κB, a key transcription factor driving inflammation and cartilage degradation. Although HSP70 is not directly implicated in the hypertrophic differentiation of chondrocytes or HIF-2α regulation at moderate pressure (e.g., 5 MPa), its induction under high mechanical stress conditions may exacerbate OA by promoting pro-inflammatory and catabolic gene expression, including MMPs. Thus, HSP70 may be a biomarker of excessive mechanical loading and a potential mediator of OA progression via stress and inflammation pathways (82). Culturing articular chondrocytes at 41°C significantly increased both HSP70 mRNA and protein expression, which coincided with improved heat stress tolerance and reduced cell death following excessive thermal exposure. HSP70 upregulation may mitigate cartilage degeneration by maintaining chondrocyte viability and cellular homeostasis under pathophysiological conditions like inflammation or mechanical overload, which are commonly seen in OA. These findings suggest that thermotherapy inducing HSP70 expression could contribute to chondroprotection and offer a novel adjunctive strategy in OA management (83).

#### 3.4.5 Response to oxidative stress

In addition, HSP70 expression is upregulated in response to oxidative stress, such as exposure to hydrogen peroxide (H_2_O_2_), and this upregulation is significantly enhanced by geranylgeranylacetone (GGA), a known HSP70 inducer. GGA treatment increased HSP70 production and reduced apoptosis in OA chondrocytes by suppressing activation of caspase 3 and 9, enzymes involved in the mitochondrial apoptotic pathway (84).

#### 3.4.6 Molecular regulation of HSP70

As an ER-resident chaperone belonging to the HSP70 family, HSPA5 is essential for overseeing protein quality control and ensuring proteostasis, functions that become particularly critical under stress conditions (85). It protects cells by stabilizing unfolded proteins and mitigating ERS (86). HSPA5 safeguards cartilage in OA by maintaining the stability of GPX4, an essential enzyme that blocks ferroptotic cell death mediated by lipid peroxidation and iron overload. However, SND1, an RNA-binding protein, binds to the 3′UTR of HSPA5 transcripts, destabilizing them and lowering HSPA5 protein levels. This suppression diminishes GPX4 stability, resulting in increased ROS production, intensified lipid peroxidation, and the aggravation of cartilage degeneration (87). The RNA-binding protein ZFP36L1 is a key contributor to OA progression through its regulation of HSP70 family members, particularly HSPA1A and HSPA1B, at the post-transcriptional level. In OA chondrocytes, ZFP36L1 expression is induced by catabolic mediators such as IL-1β, HIF-2α, and ZIP8. Once upregulated, it binds to AU-rich elements within the 3′-UTRs of HSPA1A/B transcripts, promoting their degradation. Loss or silencing of Zfp36l1 enhances HSPA1A/B expression, which in turn protects chondrocytes from apoptosis, a central mechanism of cartilage loss in OA. Notably, HSPA1A overexpression alone markedly attenuates cartilage erosion in experimental OA without influencing osteophyte formation or subchondral bone remodeling. Importantly, silencing HSPA1A in Zfp36l1-deficient mice abolishes these benefits, establishing HSPA1A as the principal mediator of the chondroprotective effects associated with ZFP36L1 suppression (88). In OA, HSP70, specifically the isoform HSPA1A (also known as Hsp72), plays a unique and contrasting role within the ERS response (89). Unlike other ER chaperones that exhibit pro-inflammatory properties, HSPA1A displays anti-inflammatory effects (90). HSPA1A suppresses NF-κB signaling and diminishes the production of pro-inflammatory cytokines. In synovial tissues from OA, its expression was notably reduced in cases of severe synovitis, most prominently in RA compared with OA and CPPA, and showed a negative correlation with both histological inflammation scores and the expression of ten ER stress–related proteins. These findings suggest that advanced synovitis is associated with an impaired anti-inflammatory capacity. Moreover, the HSPA1A/TXNDC5 ratio was inversely related to inflammatory severity, indicating that a shift toward pro-inflammatory ER proteins such as TXNDC5, at the expense of anti-inflammatory mediators like HSPA1A, may drive synovial inflammation and the proliferative activity of fibroblast-like synoviocytes (FLSs) in OA (89). HSP-70 plays a protective and regulatory role in OA of the hip by preserving cellular integrity and inhibiting apoptosis in response to joint stress. It prevents chondrocyte death by suppressing key apoptotic pathways (e.g., caspase-3, NF-κB, and cytochrome C release) and protecting cells from oxidative damage. HSP-70 expression was significantly elevated in chondral and subchondral tissues of patients with severe OA compared to controls, likely reflecting an adaptive response to joint damage. Its co-expression with Syndecan-1 suggests a joint effort in cellular repair and inflammation control. However, in cases of severe biomechanical stress, such as from obesity or trauma, HSP-70’s protective effect may be limited due to the upregulation of HSP-90, which inhibits HSP-70 and contributes to inflammation via NF-κB activation (91). Hsp70 plays a crucial cytoprotective role in chondrocytes affected by OA by suppressing apoptosis and maintaining cartilage integrity. It is significantly upregulated in OA chondrocytes, promoting the synthesis of essential extracellular matrix components such as type II collagen and proteoglycan core protein. Significantly, Hsp70 inhibits apoptosis by blocking the activation of caspase-3, a key executor of the apoptotic pathway (**Figure 4**). This protective mechanism interferes with apoptosome assembly and limits caspase-3 nuclear translocation. However, in OA conditions, SGTB overexpression negatively regulates Hsp70, reducing its protective effect and thereby enhancing caspase-dependent apoptosis (92).

### 3.5 GRP78

GRP78 (Glucose-Regulated Protein 78), or BiP as a member of the HSP70 family, is an essential ER chaperone protein that helps maintain protein-folding homeostasis by binding to misfolded or unfolded proteins and facilitating their proper folding or degradation (93). In osteoarthritic cartilage, GRP78 is upregulated as part of the protective ER stress response, primarily via the IRE1-XBP1 pathway. It is key in enhancing the cell’s capacity to manage ER stress. However, although GRP78 expression increases in OA cartilage, its expression remains localized (mainly to the upper middle zones). It does not increase significantly with cartilage degeneration, showing only a weak correlation with OA severity. In contrast, markers of the apoptotic ER stress response, such as CHOP, pPERK, and Ub, strongly correlate with cartilage damage and chondrocyte apoptosis. This imbalance, where the protective GRP78-mediated response is insufficient or diminished, while the pro-apoptotic signaling intensifies, suggests that a reduced protective response alongside an enhanced apoptotic response contributes to ER stress, induced chondrocyte apoptosis, and ECM degradation in OA cartilage (94). In OA in humans, Grp78 and Bag-1 are upregulated in chondrocytes, indicating the presence of ERS, which is likely triggered by the heightened metabolic activity required for excessive synthesis of ECM proteins, including collagen VI. Grp78, a molecular chaperone and marker of the UPR, is elevated in response to this ER stress, reflecting disrupted protein folding and processing. Bag-1, which has anti-apoptotic and cell survival functions, is upregulated throughout all cartilage zones in advanced OA, possibly as a compensatory mechanism to counteract apoptosis and promote cell survival and proliferation. Simultaneously, collagen VI is significantly upregulated and redistributed from the pericellular matrix to the interstitial space, suggesting impaired matrix organization and cell-matrix interaction (95). In knee OA, the synovial fluid contains microparticles (MPs) that present the intracellular stress proteins vimentin and GRP78 on their outer membrane, confirming that these vesicles can externalize putative autoantigens even in a largely “non-autoimmune” arthropathy. However, the proportion of vimentin^+^ and GRP78^+^ MPs in OA fluid is markedly lower than in rheumatoid arthritis. When OA-derived MPs were added to primary fibroblast-like synoviocytes cultured from OA tissue, they elicited only a modest secretory response: BAFF (B-cell activating factor) rose reliably, and smaller increases were seen in CXCL6, CCL8, and CCL15. At the same time, classical pro-inflammatory mediators such as TNF-α, IL-8, CXCL2, and CXCL5 were either unchanged or minimally induced. This subdued profile contrasts with the broad, high-level cytokine/chemokine burst triggered by rheumatoid MPs, indicating that although OA MPs can engage synoviocytes via surface vimentin and GRP78, the downstream activation is limited, reflecting both their lower abundance and the comparatively restrained inflammatory environment of osteoarthritic joints (96).

#### 3.5.1 GRP78’s role in mechanical stress

Mechanical stress from abnormal dental occlusion, such as unilateral anterior crossbite (UAC), induces calcium overload in the ER of articular chondrocytes in the temporomandibular joint (TMJ) (97). This calcium imbalance disrupts ER homeostasis, accumulating unfolded proteins, a state known as ER stress (98). In response, the ER chaperone GRP78 (HSPA5), a critical sensor and marker of ER stress, becomes activated to attempt to restore protein-folding equilibrium (99). Persistent mechanical loading sustains this ER stress response, promoting prolonged GRP78 expression and activating downstream ER stress-apoptosis signaling pathways mediated primarily by phosphorylated EIF2AK3 (100). Concurrently, mechanical stress initially triggers protective autophagy; however, sustained stress shifts the cellular response towards apoptosis due to activation of the mTORC1 pathway. Thus, mechanical stress activates GRP78 as part of a broader ER stress response, ultimately contributing to the progression of osteoarthritic lesions in the TMJ by tipping the balance from chondrocyte survival toward apoptosis (97).

#### 3.5.2 Effect of pollution on GRP78 in OA

Pollution, particularly through environmental contaminants such as pesticides, can activate the ERS marker GRP78, contributing to the development or exacerbation of OA. Exposure to pollutants like thiram, a widely used agricultural fungicide, triggers cellular stress by disrupting protein folding mechanisms within the ER, causing an accumulation of misfolded or unfolded proteins (101). This stress activates GRP78, a molecular chaperone and key regulator of the UPR, which is crucial in maintaining cellular proteostasis (102). However, chronic pollution exposure persistently elevates GRP78, signaling sustained ER stress and harmful downstream effects, such as impaired calcium homeostasis and mitochondrial dysfunction (103). These disturbances exacerbate inflammation by activating the NLRP3 inflammasome pathway, contributing to joint tissue degradation, cartilage loss, and worsening OA symptoms (101). T-2 toxin, a cytotoxic mycotoxin produced by Fusarium species, induces chondrocyte apoptosis primarily through ERS, with GRP78 acting as a central mediator in this pathway. Upon exposure to T-2 toxin, GRP78 is upregulated, indicating ER dysfunction and the UPR activation. This stress response leads to the activation of the PERK-eIF2α-ATF4-CHOP signaling cascade, which ultimately triggers apoptotic mechanisms. Specifically, CHOP promotes pro-apoptotic signals, while ER-specific caspase-12 is also activated, further amplifying apoptosis in chondrocytes. These molecular events disrupt ECM homeostasis, contributing to cartilage degradation typical of OA (104).

#### 3.5.3 GRP78 role in extracellular matrix degradation

In OA, ECM degradation is a key pathological feature that contributes to cartilage deterioration and joint dysfunction (105). The ECM, primarily composed of type II collagen and aggrecan, is essential for maintaining cartilage structure and function (106). Inflammatory stimuli such as IL-1β upregulate the expression of ECM-degrading enzymes, including matrix metalloproteinases (MMP3, MMP-13) and aggrecanases (ADAMTS-4, ADAMTS-5), leading to ECM breakdown (107). GRP78 is upregulated in OA and promotes this degradation process (108). It enhances ERS signaling pathways, further triggering inflammation and chondrocyte apoptosis, exacerbating ECM breakdown. Silencing PLXNC1 downregulated GRP78 expression, thereby reducing ERS signaling and the expression of ECM-degrading enzymes, ultimately preserving ECM integrity in OA models (109).

#### 3.5.4 Response to hypoxia

Mechanical and hypoxia stress play pivotal roles in regulating GRP78 expression in OA, particularly in the TMJ. GRP78 is significantly upregulated in chondrocytes when exposed to excessive mechanical loading (e.g., 20% cyclic strain) or severe hypoxia (e.g., 1% O_2_), surpassing the cellular adaptive threshold. This overactivation of ERS leads to chondrocyte apoptosis and contributes to OA-like cartilage degradation. *In vivo*, mechanical stress not only increases GRP78 levels but also induces hypoxia, suggesting a compounding effect. The ERS inhibitor Salubrinal suppresses GRP78 expression under mechanical and hypoxic stress, thereby reducing apoptosis and alleviating cartilage damage (110).

#### 3.5.5 GRP78’s role in disease stage in OA

In OA, particularly primary knee OA (pkOA), the disease stage significantly influences the levels of GRP78 in the synovial fluid (SF). GRP78 levels progressively increased with advancing Kellgren–Lawrence (K–L) radiographic grades, indicating a strong positive correlation between disease severity and ER stress marker expression. Specifically, patients with higher K–L grades (indicative of more severe joint degeneration) exhibited significantly elevated GRP78 levels in the SF compared to those with milder OA. This trend suggests that GRP78 may play a role in cartilage degeneration and joint damage as OA progresses, potentially serving as a local biomarker of disease advancement and ER stress–related cellular dysfunction in chondrocytes (109).

#### 3.5.6 GRP78 in the onset of OA

At the onset of OA, particularly following joint destabilization (such as through DMM surgery in mice), articular chondrocytes experience elevated ERS. This is evidenced by early upregulation of GRP78/BiP, a key ER chaperone and canonical marker of ER stress. This suggests that ER stress is not merely a byproduct of OA but an early and localized cellular response to mechanical or structural joint damage. The elevated expression of GRP78 reflects activation of the UPR, aiming to restore proteostasis; however, if unresolved, it may also prime cells for apoptosis. Thus, GRP78 is both an indicator of stress and a potential modulator of chondrocyte fate during OA onset (111).

#### 3.5.7 Molecular regulation of GRP78 in OA

GRP78 (also called BiP) serves as the master sentinel of the endoplasmic-reticulum stress response in chondrocytes (112). When misfolded proteins accumulate, GRP78 expression rises, and the chaperone disengages from the three UPR sensors, PERK, IRE1α, and ATF6, while a fraction of the protein relocates to the cell surface (113).

##### 3.5.7.1 Interaction with PI3K/Akt pathway

**Figure 5:**
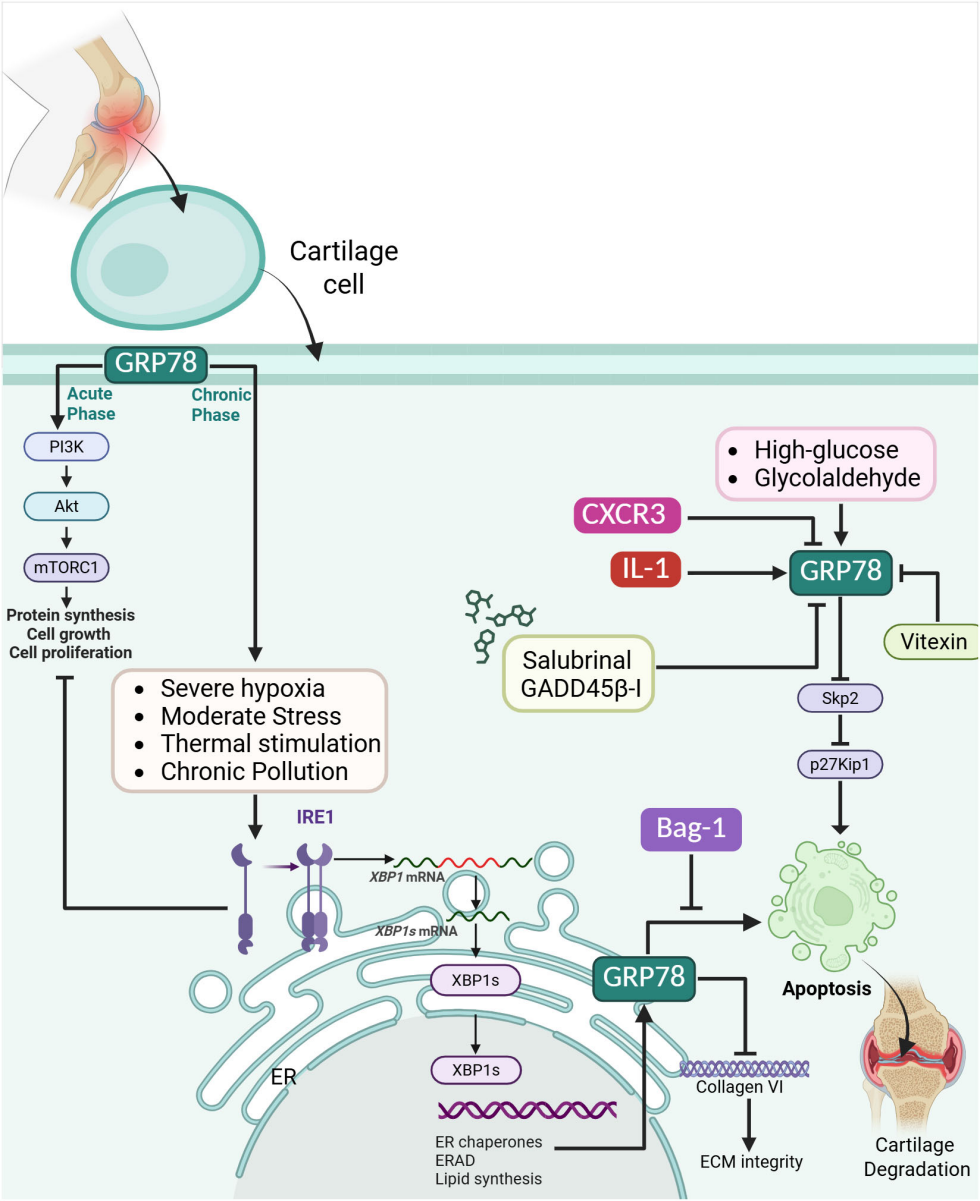
GRP78-mediated ER stress response in osteoarthritis. GRP78 is upregulated in chondrocytes during acute stress via the PI3K–Akt–mTORC1 pathway to support protein synthesis, proliferation, and survival. At the same time, persistent stressors such as hypoxia, high glucose, glycolaldehyde, and pollution drive chronic ER stress, apoptosis, and ECM degradation. GRP78 activates the IRE1–XBP1 axis, enhancing ER chaperone expression, ERAD, and lipid synthesis, but excessive signaling shifts toward apoptosis through Skp2–p27Kip1 suppression and CXCR3–IL-1–mediated pathways. Pharmacological inhibitors (Salubrinal, GADD45β-I) and natural compounds (Vitexin) attenuate GRP78 expression, protecting against ER stress-induced apoptosis. Bag-1 upregulation counteracts apoptosis by promoting survival, while GRP78 maintains ECM integrity through regulation of collagen VI. Excessive or prolonged GRP78 activation, however, contributes to cartilage degeneration by tipping the balance from adaptive proteostasis toward apoptotic signaling.

At that membrane locale, GRP78 acts as a signaling hub: its extracellular domain binds stress-related ligands such as activated α2-macroglobulin or thrombospondin, and the resulting conformational change recruits the p85 regulatory subunit of phosphoinositide-3-kinase (114). PI3K, in turn, generates phosphatidylinositol-3,4,5-trisphosphate, which docks and activates Akt through Thr308/Ser473 phosphorylation, propagating the classical PI3K/Akt cascade (**Figure 5**) (115). Transient activation helps chondrocytes survive by boosting anabolic gene expression and dampening inflammatory signaling. Still, persistent or excessive GRP78 up-regulation, as seen in mechanically overloaded or LPS-challenged temporomandibular cartilage, sustains Akt activity while simultaneously driving the ATF4-CHOP and caspase-12 arms of the UPR, tipping the balance toward apoptosis and matrix degradation. Thus, GRP78 functions upstream of, and is a key regulator of, PI3K/Akt signaling in OA, orchestrating the switch between adaptive cell survival and stress-induced chondrocyte death (114).

##### 3.5.7.2 Effects on mTOR

GRP78 is the stress-sensing chaperone that first responds to P2X7-driven Ca²^+^ overload in the endoplasmic reticulum; by dissociating from its three unfolded-protein-response partners, it simultaneously licenses the IRE1-, PERK-, and ATF6-arms of the UPR. Moderate stress favors IRE1 signaling: IRE1 suppresses mTOR activity, allowing Beclin-1/LC3B-dependent autophagic flux (confirmed by LC3B–LAMP2 colocalization) to clear damaged proteins and restore homeostasis. As mechanical or ATP stimulation intensifies, GRP78 remains elevated, but the balance tips, IRE1 output wanes, mTOR rephosphorylates, and activated mTOR switches on PERK. PERK then blocks autophagosome-lysosome fusion and, through CHOP and caspase-12, drives ER-stress apoptosis (116).

##### 3.5.7.3 High glucose overload

Under prolonged high-glucose conditions, chondrocytes experience heightened endoplasmic-reticulum stress signaled by an increase in the master chaperone GRP78. GRP78-driven unfolded-protein response cascades suppress expression of the F-box protein Skp2, the E3-ubiquitin-ligase subunit that normally targets the cyclin-dependent-kinase inhibitor p27Kip1 for degradation. As Skp2 levels fall, p27 accumulates, enforcing G1 arrest and sharply reducing chondrocyte proliferation and extracellular-matrix synthesis (collagen II, aggrecan). Pharmacological alleviation of ERS with 4-PBA restores Skp2 and lowers p27, while exogenous Skp2 over-expression rescues proliferation even when GRP78-mediated stress remains high, confirming that the Skp2/p27 axis is the critical downstream effector linking GRP78-triggered ER stress to growth suppression in high-glucose, diabetic-like cartilage (117).

##### 3.5.7.4 Interaction with vitexin

Vitexin regulates GRP78 expression in OA by alleviating ERS in chondrocytes, a key contributor to cartilage degeneration. GRP78, a central marker and regulator of ER stress, is significantly upregulated in OA conditions, especially in response to inflammatory stimuli like interleukin-1β (IL-1β) or thapsigargin (TG), a known ER stress inducer. In both *in vitro* and *in vivo* models, vitexin treatment led to a marked reduction in GRP78 levels, suggesting that it mitigates ER stress and its downstream apoptotic signaling. By downregulating GRP78, vitexin helps preserve chondrocyte viability, reduces apoptosis, and protects cartilage from OA-related degeneration (118).

##### 3.5.7.5 Interaction with IL-1β

In OA, GADD45β-I, a selective inhibitor of MKK7, attenuated ER stress in chondrocytes by downregulating GRP78, a key ER chaperone upregulated during stress conditions. Upon IL-1β exposure, GRP78 expression markedly increased, indicating heightened ER stress and contributing to chondrocyte apoptosis. However, treatment with GADD45β-I significantly reduced GRP78 levels, as evidenced by immunostaining and fluorescence quantification, alleviating ER stress. This regulatory effect of GADD45β-I on GRP78 was accompanied by increased chondrocyte survival and reduced apoptosis, suggesting that suppression of GRP78-mediated ER stress is a pivotal mechanism by which GADD45β-I confers protection against IL-1β-induced cytotoxicity in OA (119).

##### 3.5.7.6 Interaction with sex hormones

Estradiol, the primary female sex hormone, plays a protective role in cartilage health by reducing ERS and ERS-induced apoptosis in chondrocytes, the cells responsible for maintaining cartilage. Physiological concentrations of estradiol effectively lower the expression of ER stress markers (such as calnexin and BiP) and decrease apoptosis in chondrocytes, particularly under stress conditions induced by ER stressors like thapsigargin or the absence of the chaperone ERp57. In both *in vitro* cell models and vivo mouse models, estradiol significantly reduced ER stress and apoptosis, which are key contributors to OA progression. As a result, female mice, likely due to the presence of estradiol, exhibited less severe age-related cartilage degeneration compared to males (120).

##### 3.5.7.7 Interaction with CXCR3

Chemokine receptor CXCR3 plays a regulatory role in chondrocyte apoptosis by modulating nitric oxide (NO)-induced ERS. Specifically, CXCR3 expression was significantly elevated in osteoarthritic cartilage and in chondrocytes exposed to inflammatory stimuli such as IL-1β and sodium nitroprusside (SNP, a NO donor). The knockdown of CXCR3 via siRNA reduced NO levels in chondrocytes and attenuated apoptosis induced by SNP, but not by IL-1β. Mechanistically, CXCR3 silencing led to downregulation of ER stress markers GRP78 and CHOP, suggesting that CXCR3 exacerbates NO-mediated ER stress and promotes chondrocyte apoptosis by upregulating GRP78 (121).

##### 3.5.7.8 Effects of advanced glycation end products on GRP78

Intracellular accumulation of advanced glycation end products (AGEs) is critical in OA by disrupting the endoplasmic reticulum chaperone protein GRP78 function. In chondrocytes exposed to glycolaldehyde (GA), an AGE precursor, AGEs accumulated intracellularly and co-localized with GRP78, leading to its modification and aggregation into high-molecular-weight complexes. This AGE-mediated modification likely impaired GRP78’s chaperone function, triggering ER stress and activating the UPR. As stress progressed, the signaling shifted from a protective to a pro-apoptotic response marked by upregulation of CHOP, a key ER stress-induced apoptosis factor. Inhibiting AGE formation with aminoguanidine reduced GRP78 modification, ER stress markers, and chondrocyte apoptosis (**Figure 5**) (122).

### 3.6 HSP90

HSP90 is a molecular chaperone that stabilizes and assists in properly folding a wide range of client proteins, many of which are involved in cellular stress responses, inflammation, and apoptosis (123). In OA, HSP90 acts as a damage-associated molecular pattern (DAMP), meaning stressed or necroptotic chondrocytes release it and trigger inflammatory responses in the joint environment (124).

#### 3.6.1 Necroptosis regulation

HSP90 was identified as one of the DAMPs released by necroptotic condylar chondrocytes in temporomandibular joint osteoarthritis (TMJOA). Proteomic analysis of necroptotic cell supernatants revealed elevated levels of SDC4, HSP90, and S100A13, with ELISA confirming significant increases in HSP90 after necroptosis induction. *In vivo*, HSP90-positive chondrocytes were abundant in TMJOA cartilage but decreased markedly after treatment with RIPK3 or MLKL inhibitors, suggesting its release is linked to necroptotic cell death (125).

#### 3.6.2 Synovial fibrosis

In OA, downregulation of hsa-miR338-3p leads to upregulation of TRAP-1, a member of the HSP90 family, which plays a pivotal role in promoting synovial fibrosis (126). TRAP-1 enhances the TGF-β/Smad signaling pathway by facilitating Smad2/3 phosphorylation and Smad4 complex formation, ultimately activating fibroblast-like synoviocytes (FLSs) and increasing the expression of fibrosis markers such as vimentin, collagen I, and TIMP1. This fibrotic activation contributes to joint stiffness and adhesion in OA patients. Overexpression of hsa-miR338-3p suppresses TRAP-1 expression, downregulates the TGF-β/Smad pathway, and thus mitigates synovial fibrosis, highlighting a potential therapeutic axis involving HSP90/TRAP-1 modulation via hsa-miR338-3p (127).

#### 3.6.3 Cellular stress response

Hsp90 inhibition protects against biomechanically induced OA by shifting the cellular stress response in chondrocytes toward a protective phenotype. Strenuous mechanical loading increases Hsp90 levels, which activate NF-κB signaling and suppress Hsp70 expression, contributing to cartilage degradation, chondrocyte apoptosis, and OA progression. Inhibiting Hsp90 with BIIB021 disrupts this pathway, leading to functional inactivation of Hsp90 and disinhibition of heat shock factor 1 (HSF-1), increasing Hsp70 expression. Elevated Hsp70 enhances chondrocyte survival and extracellular matrix integrity by maintaining sulfated glycosaminoglycan (sGAG) levels, reducing cartilage degradation, and improving tissue resilience to mechanical stress. Additionally, Hsp90 inhibition preserves subchondral bone structure and initially reduces macrophage-driven inflammation in the joint (128). Inhibition of Hsp90β in human chondrocytes significantly reduces nitric oxide (NO) production and NO-induced apoptosis, suggesting a protective role against OA-related cellular stress. The chaperone Hsp90β, upregulated in OA and further increased by proinflammatory cytokines (IL-1β, TNF-α) and NO donors (NOC-12, SNP), enhances NO synthesis, likely through its interaction with inducible nitric oxide synthase (iNOS). Treatment with Hsp90β inhibitors (Geldanamycin and Novobiocin) led to a dose-dependent decrease in IL-1β-induced NO levels and mitigated cytokine-induced morphological changes without compromising cell viability. Furthermore, pharmacological inhibition and siRNA-mediated knockdown of Hsp90β markedly reduced apoptosis triggered by NO donors, confirming a direct role of Hsp90β in NO-mediated chondrocyte death (129).

#### 3.6.4 Response to hypoxia

Under hypoxic conditions, expression of TRAP1, a mitochondrial chaperone and member of the HSP90 family, was significantly upregulated in OA chondrocytes compared to normal chondrocytes. This upregulation was evident at both the protein and mRNA levels, as confirmed by proteomics, real-time PCR, and immunofluorescence analyses. TRAP1 was particularly abundant in the deep layer of OA cartilage, which naturally experiences the lowest oxygen tension. Unlike general metabolic enzymes that tended to decrease under hypoxia (indicating reduced metabolic activity), TRAP1’s increase suggests a protective, adaptive response to prolonged low-oxygen stress, possibly contributing to mitochondrial stability and chondrocyte survival. This makes TRAP1 a potential player in OA pathogenesis and cellular stress management under hypoxia (130).

#### 3.6.5 Comparison of HSP90α function in OA and RA

Hsp90α gene expression was significantly higher in synovial tissues of RA patients compared to those with OA, suggesting a more prominent role of Hsp90α in inflammatory joint disease than in non-inflammatory conditions like OA. However, in OA synovial tissues, Hsp90α expression was relatively lower, indicating that Hsp90α is not substantially upregulated in OA and likely does not contribute significantly to OA pathogenesis. This differential expression highlights Hsp90α’s potential utility as a diagnostic marker to distinguish RA from OA rather than indicating a key role in OA itself (131).

#### 3.6.6 Molecular regulation of HSP90

**Figure 6:**
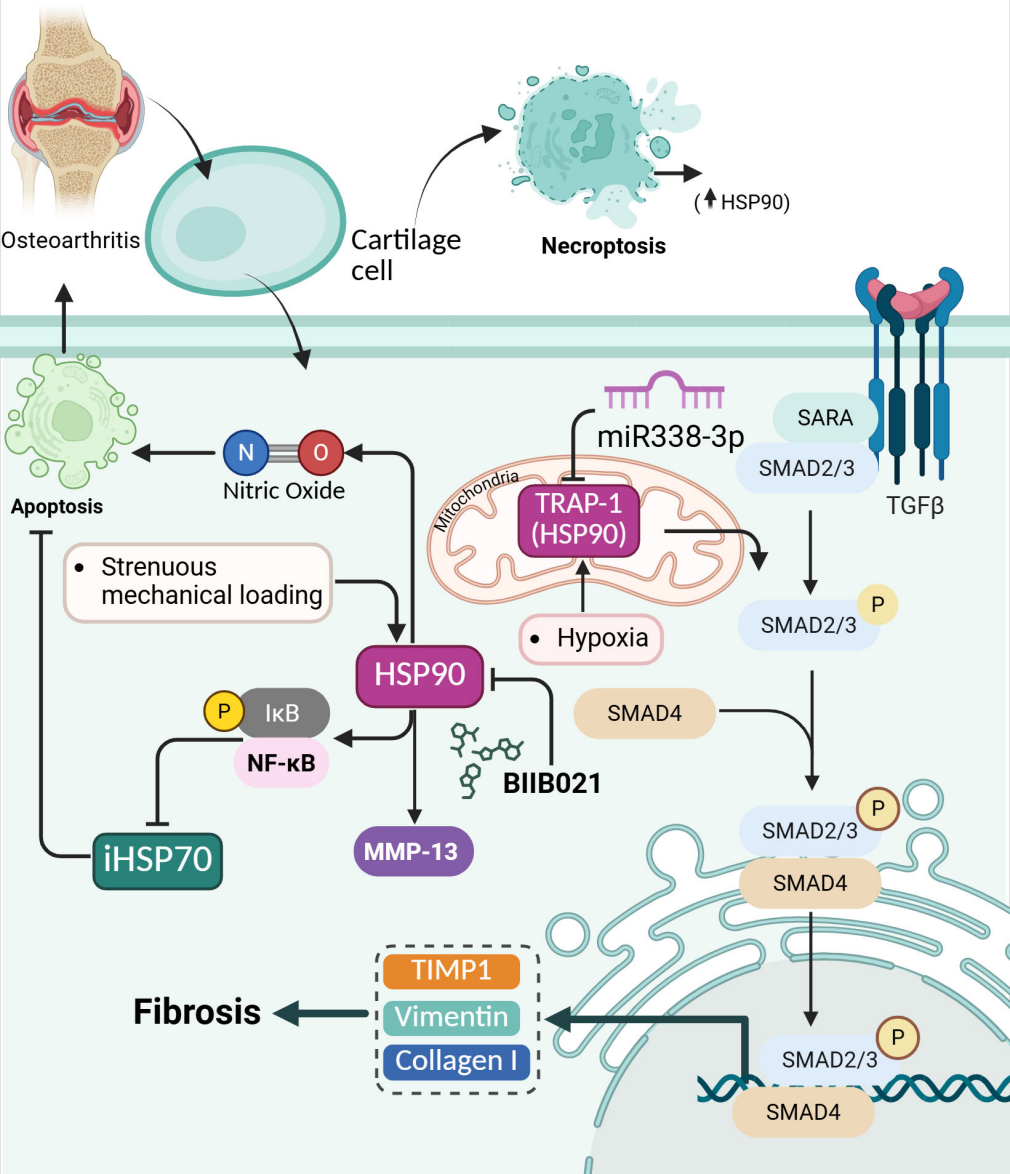
Role of HSP90 in chondrocyte stress responses, apoptosis, and fibrosis in osteoarthritis. HSP90 is upregulated by strenuous mechanical loading, hypoxia, nitric oxide, and inflammatory stimuli, where it promotes NF-κB activation, MMP-13 expression, and apoptosis, while suppressing iHSP70. TRAP-1, the mitochondrial isoform of HSP90, is elevated under hypoxia and enhances TGFβ–Smad2/3–Smad4 signaling, driving synovial fibrosis through increased expression of vimentin, collagen I, and TIMP1. Downregulation of miR338-3p further increases TRAP-1, amplifying fibrotic signaling. Necroptotic chondrocytes release HSP90 as a DAMP, propagating inflammation. Pharmacological inhibition of HSP90 with BIIB021 restores HSP70 expression, reduces NF-κB activation, protects cartilage integrity, and mitigates fibrosis, highlighting HSP90 as a key pathogenic factor and therapeutic target in OA.

In contrast to HSP90α, HSP90β demonstrates a more defined functional involvement in osteoarthritis pathophysiology. Hsp90β acts as a negative regulator of MMP-13 expression in human OA chondrocytes, particularly in the low-expressing (L-OA) chondrocyte phenotype. Mass spectrometry identified Hsp90β as a component of the protein complex bound to the AGRE site on the MMP-13 promoter, where it likely exerts its repressive function indirectly by interacting with other regulatory proteins. Silencing Hsp90β using siRNA significantly increased both basal and IL-1β-induced MMP-13 mRNA and protein levels, without affecting upstream IL-1β-induced MAPK or NF-κB signaling. IL-1β downregulated Hsp90β expression and protein production, which indirectly enhances MMP-13 expression by reducing this inhibitory influence (**Figure 6**) (132). In equine articular chondrocytes, Hsp90 supports IL-1β-induced upregulation of matrix metalloproteinase 13 (MMP13), a key enzyme in cartilage degradation. Inhibition of Hsp90 by geldanamycin blocks both IGF-1-mediated anabolic responses and IL-1β-induced catabolic responses, but at different sensitivity thresholds, MMP13 suppression occurs at lower geldanamycin concentrations than those required to inhibit COL2A1 expression (133).

## 4 Treatment strategies targeting HSPs in osteoarthritis

**Figure 7:**
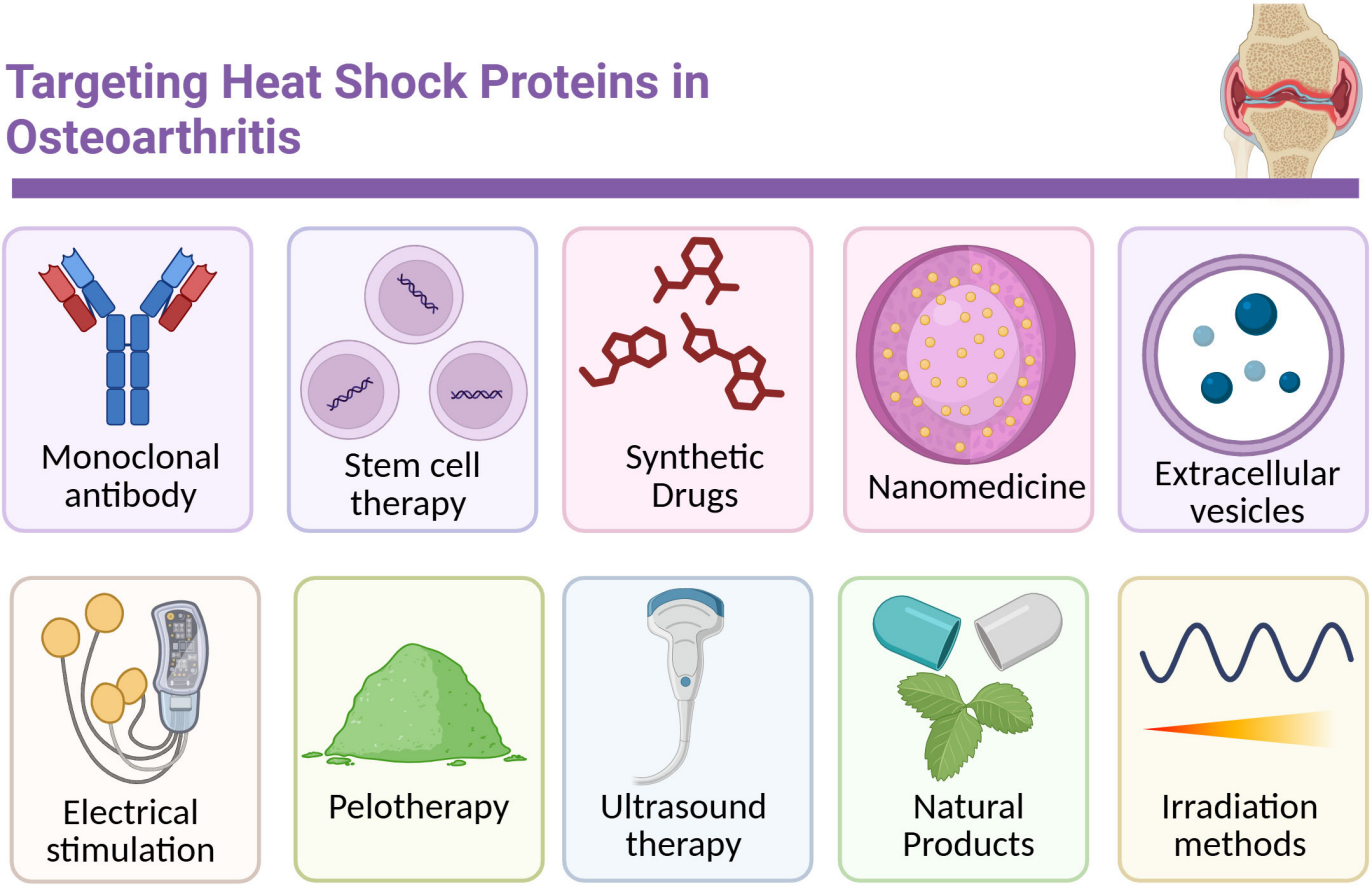
Overview of treatment options targeting HSPs in OA treatment.

Targeting HSPs represents a compelling therapeutic avenue in OA, as both are central regulators of proteostasis, cell survival, and inflammatory balance within joint tissues (8, 134). HSPs, particularly chaperones such as HSP70 and GRP78, assist in protein quality control, protect against oxidative and mechanical stress, and inhibit apoptosis in joint cells. Enhancing protective HSP activity while mitigating maladaptive ER stress could preserve chondrocyte viability, maintain extracellular matrix integrity, and slow disease progression, making these pathways attractive targets for developing disease-modifying OA therapies [Reviewed by (135)]. This section outlines therapeutic strategies aimed at targeting HSPs in osteoarthritis (**Figure 7**, **Supplementary Table 1**).

### 4.1 Synthetic drugs

Synthetic OA drugs primarily include non-steroidal anti-inflammatory drugs (NSAIDs) like ibuprofen and celecoxib, and corticosteroids such as hydrocortisone and dexamethasone (136). These drugs work by inhibiting key inflammatory pathways, particularly the cyclooxygenase (COX) and NF-κB signaling pathways, which produce pro-inflammatory mediators like TNF-α and IL-1β (137). Synthetic drugs reduce inflammation, pain, and swelling associated with OA by blocking these pathways (138). However, while effectively alleviating symptoms, they do not halt or reverse cartilage degradation. Long-term use of synthetic drugs may also lead to serious side effects such as gastrointestinal issues, increased infection risk, mood changes, or bone loss, which limits their use for chronic treatment (139). High-molecular-weight hyaluronic acid (HMW-HA), typically ranging from 1000 to 2000 kDa, is a therapeutic agent commonly used in OA treatment for its viscoelastic and anti-inflammatory properties (140). In the context of OA, where synovial inflammation and macrophage dysregulation contribute to disease progression, HMW-HA significantly suppresses IL-1β-induced inflammatory responses in synoviocytes (141). It achieves this by downregulating the expression of GRP78, a key marker of ERS, inhibiting activation of the NF-κB pathway, a central mediator of proinflammatory signaling. This suppression decreases proinflammatory cytokines (such as IL-6 and PGE2) and shifts macrophage polarization from the proinflammatory M1 phenotype to the anti-inflammatory M2 phenotype (142, 143). The effect is enhanced when HMW-HA treatment is combined with GRP78 knockdown, highlighting the GRP78-NF-κB axis as a crucial target of HMW-HA’s anti-inflammatory action in OA (144). VA692 is a novel, selective COX-2 inhibitor derived from the 1,5-diarylpyrrole scaffold with dual anti-inflammatory and nitric oxide-releasing properties. It has demonstrated strong anti-inflammatory, antioxidant, anti-nociceptive, and chondroprotective effects in both IL-1β-stimulated human chondrocyte cell lines (T/C-28a2) and primary OA chondrocytes. Compared to celecoxib, VA692 more effectively modulates inflammation by suppressing COX-2, IL-1β, IL-6, IL-8 expression, and PGE2 release, reducing oxidative stress and apoptosis. Notably, proteomic analysis revealed that VA692 downregulates key heat shock proteins, specifically HSP90α, HSP90β, and HSP70 (HSP7C), which are typically elevated in OA and contribute to cartilage degeneration by promoting MMP expression, NO synthesis, and COX-2 production. By inhibiting these HSPs, VA692 may restore cellular homeostasis and reduce catabolic responses in chondrocytes, suggesting its potential role in modulating HSP-mediated stress responses and preserving cartilage integrity in OA (145). In addition, ITZ-1 is a novel small molecule identified as a client-selective inhibitor of Hsp90 that exhibits chondroprotective effects in OA. It exerts its action primarily by binding to the C-terminal domain of Hsp90, unlike most known Hsp90 inhibitors that target the N-terminal ATP-binding site. By binding to Hsp90, ITZ-1 selectively induces degradation of the client protein Raf-1, suppressing IL–1β–induced ERK activation, inhibiting MMP-13 production, a key enzyme responsible for cartilage degradation. Moreover, ITZ-1 promotes dissociation of the HSF1/Hsp90 complex, thereby activating HSF1 and significantly upregulating cytoprotective Hsp70 without causing widespread degradation of other Hsp90 client proteins, minimizing cytotoxicity (146). Likewise, MG132 is a reversible proteasome inhibitor that exerts chondroprotective effects in OA by inducing Hsp70, which plays key roles in cell survival and stress response (78). In OA, excessive chondrocyte apoptosis contributes to cartilage degradation (147). MG132, when administered intra-articularly in a rat model of OA, significantly upregulates Hsp70 expression in chondrocytes without causing chondrotoxicity (148). Hsp70 overexpression mitigates chondrocyte apoptosis by acting as a molecular chaperone, preserving mitochondrial integrity, inhibiting caspase activation, and suppressing inflammatory signaling pathways such as NF-κB (149). This dual anti-apoptotic and anti-inflammatory action helps maintain cartilage integrity and reduces synovial inflammation, suggesting that MG132 could be a promising disease-modifying agent for OA by enhancing chondrocyte resilience to stress and cytokine-mediated damage (148). Lastly, 4-Phenylbutyric acid (4-PBA), a chemical chaperone and ER stress inhibitor, protects cartilage in OA by modulating HSP-mediated stress responses. It downregulates GRP78, CHOP, pro-inflammatory cytokines (TNF-α, IL-1β), and pro-apoptotic markers (Bax, Caspase-3), while upregulating Bcl-2, thereby reducing ER stress, inflammation, and apoptosis (150).

### 4.2 Nanomedicine

Nanoparticles have emerged as promising carriers for OA therapy due to their ability to enhance intra-articular drug retention, improve tissue targeting, and provide controlled or stimuli-responsive drug release. By encapsulating anti-inflammatory agents, antioxidants, growth factors, or nucleic acids, nanoparticles can protect therapeutic molecules from enzymatic degradation and deliver them directly to chondrocytes, synoviocytes, or subchondral bone. However, their clinical translation remains limited by pharmacokinetic and biocompatibility hurdles. Rapid synovial and lymphatic clearance, poor penetration through the dense avascular cartilage, and degradation in the inflamed joint environment all shorten their residence time and efficacy (151–153). Light-inducible hybrid nanomedicine is a multifunctional nanosystem that releases therapeutic agents upon near-infrared (NIR) stimulation. In OA treatment, gold nanocages (AuNCs) loaded with diacerein (DIA) and NGF-targeted siRNA (siNGF) are coated with a cationic polymer (PBAE) for siRNA delivery and a thermo-responsive lipid shell (lauric/stearic acid) for light-triggered release. After intra-articular injection, NIR-induced photothermal heating of AuNCs melts the lipid layer, enabling on-demand release of DIA and siNGF. This dual therapy reduces ROS and NGF expression, alleviates inflammation and pain, and induces HSP70 expression, which protects chondrocytes by stabilizing proteins, preventing apoptosis, and preserving cartilage matrix (151). A multifunctional nanozyme (MPMP) is designed to mimic antioxidases (SOD, CAT) and hyaluronan synthase (HAS). Its MoS_2_ core, coated with Mg²^+^-doped polydopamine and zwitterionic polymers, scavenges ROS/RNS and generates oxygen, alleviating oxidative stress in osteoarthritic joints. Concurrently, photothermal Mg²^+^ release activates HAS-like activity and upregulates HSP70, which enhances antioxidant defenses, suppresses pro-inflammatory pathways (NF-κB, IL-17), and promotes mesenchymal stem cell chondrogenesis and HA synthesis. This dual action protects cartilage and stimulates regeneration, restoring joint homeostasis and providing a promising OA therapy (154).

### 4.3 Extracellular vesicles

Extracellular vesicles (EVs) are nano-sized, lipid bilayer-enclosed particles secreted by cells that carry bioactive molecules such as proteins, RNA, and lipids, enabling intercellular communication (155, 156). In the context of OA, EVs, particularly those derived from cartilage-derived progenitor cells (CPCs) and bone marrow mesenchymal stromal cells (BM-MSCs), can mediate tissue repair by promoting chondrocyte proliferation, migration, and anabolic gene expression (157, 158). One key mechanism by which EVs may contribute to OA treatment is HSPs, especially HSP70, which are commonly enriched in EVs and act as molecular chaperones that assist in protein folding, protect cells from stress, and modulate immune responses. While BM-MSC EVs tend to contain higher levels of HSP70, CPC EVs, despite having lower HSP70, still enhance meniscal cell viability and repair, possibly by offering a more balanced, less stress-inducing signaling environment (159).

### 4.4 Monoclonal antibody

Monoclonal antibody 9B8 is a novel therapeutic antibody targeting the HSP90α, developed by immunizing BALB/c mice with T3A-A3 cells (160). In OA, HSP90 is critical in stabilizing hypoxia-inducible factor 1-alpha (HIF-1α), a key regulator of glycolytic metabolism in hypoxic chondrocytes (161). By binding to and inhibiting HSP90α, 9B8 disrupts the proper folding and stabilization of HIF-1α, thereby suppressing downstream genes involved in glycolysis (such as PFKP and ENO2) and inflammation (e.g., TNF-α and IL-6). This results in decreased chondrocyte catabolism, improved extracellular matrix synthesis (COL2A1, ACAN), and reduced inflammatory damage. In both *in vitro* chondrocyte models and *in vivo* rat ACLT-induced OA models, 9B8 was shown to attenuate cartilage degradation and subchondral bone destruction, highlighting its potential as a disease-modifying treatment for OA via inhibition of HSP90-mediated glycolytic and inflammatory pathways (160).

### 4.5 Photosensitizers and microwave irradiation

Photosensitizers are light-activated compounds that absorb near-infrared (NIR) radiation and convert it into heat, enabling localized thermal stimulation of deep tissues like articular cartilage (162, 163). In OA therapy, intra-articular photosensitizers like indocyanine green (ICG) can be activated by NIR light to safely raise joint temperature to ~40°C. This mild hyperthermia induces HSP70 in chondrocytes, which prevents apoptosis and promotes anabolic matrix components (ACAN, SOX9). Thus, photothermal stimulation provides a minimally invasive strategy to protect cartilage and slow OA progression via HSP70-mediated mechanisms (164). Hyperthermia is being investigated as a non-invasive OA treatment that targets cartilage degeneration by inducing Hsp70. As a cytoprotective protein, Hsp70 inhibits chondrocyte apoptosis, promotes proteoglycan and type II collagen synthesis, and supports cartilage metabolism. Deep-heating methods, such as microwaves, can upregulate Hsp70 and improve cartilage integrity in OA models, though efficacy depends on heat intensity, duration, and frequency, which remain to be optimized. Combining hyperthermia with pharmacological Hsp70 inducers (e.g., glutamine, curcumin, GGA, MG132) may further enhance chondroprotection, offering a cost-effective, minimally invasive strategy to slow OA progression by supporting chondrocyte survival and matrix repair (165). Microwave (MW)-induced thermotherapy is a promising non-invasive approach for OA, as it elevates intra-articular temperature in a dose-dependent manner, with 40 W identified as optimal for stimulating cartilage metabolism without damage. MW heating markedly increased HSP70 expression, particularly at 8–24 h, which correlated with enhanced proteoglycan (PG) and type II collagen (Col II) expression. Quercetin, an HSP70 inhibitor, suppressed PG induction, confirming HSP70’s role in matrix regulation. Interestingly, Col II upregulation was HSP70-independent, suggesting the involvement of other stress proteins such as HSP42 (165). Combined MW irradiation and intra-articular glutamine (Gln) synergistically upregulate HSP70 in articular cartilage, enhancing chondrocyte protection and matrix metabolism in a rat OA model. MW at 40 W for 20 min raised joint temperature to ~38.3°C, sufficient to induce HSP70 without tissue damage. While Gln alone had no effect, its combination with MW markedly increased HSP70 and aggrecan expression, reflecting enhanced anabolic activity. Inhibition of HSP70 by quercetin or siRNA reduced aggrecan and abolished the protective effects. Histology further confirmed preserved cartilage and reduced OA severity in the MW + Gln group compared to controls (166). Likewise, laser irradiation applies focused light energy to tissues, modulating cellular responses. In OA, it upregulates HSPs, particularly HSP70, which stabilize proteins, protect chondrocytes from apoptosis, and reduce inflammation. Increased HSP70 expression in articular cartilage has been shown to alleviate joint inflammation, limit cartilage degradation, and improve OA symptoms (167).

### 4.6 Stem cell therapy

Stem cell therapy in OA is an emerging regenerative treatment aimed at addressing the root cause of the disease, articular cartilage degeneration, rather than merely alleviating symptoms. Among the various stem cell types, mesenchymal stem cells (MSCs), particularly those derived from bone marrow (BMSCs) and adipose tissue (ASCs), are most commonly used due to their abundance, immunomodulatory properties, and ability to differentiate into chondrocytes (168). Articular cartilage stem cells (ACSCs) are a specialized population of cartilage-resident progenitor cells possessing self-renewal and multipotent differentiation capabilities, particularly into chondrocytes (169). These cells contribute to cartilage repair by homing to injury sites, modulating the microenvironment, and promoting matrix regeneration (170). In OA, ACSCs exert a therapeutic effect by attenuating ERS in chondrocytes, a key driver of apoptosis in cartilage degeneration (7). Mechanistically, ACSCs influence the PERK–eIF2α–ATF4 pathway, a significant branch of the UPR, activated during ERS. By modulating this pathway, ACSCs enhance the expression of GRP78 and other cytoprotective factors (e.g., TMEM119, BMP6), thereby promoting protein homeostasis, reducing apoptosis (as evidenced by improved Bcl-2/Bax ratios and decreased caspase-3 activation), and supporting chondrocyte survival. Although ACSCs upregulate PERK expression, their overall effect appears to balance protective stress responses with inhibition of terminal apoptosis, making them promising candidates for OA therapy targeting HSP- and PERK-mediated mechanisms (171). Pelleted bone marrow-derived mesenchymal stem cells (hBMMSCs) form aggregates that create a protective microenvironment, enhancing survival under stress compared to dispersed suspensions. In OA, these pellets show greater metabolic stability and lower expression of apoptotic and inflammatory genes. HSP70 mediates their cytoprotective effects by stabilizing proteins, suppressing apoptosis, and promoting resilience under mild heat stress. This response supports hBMMSC survival and function in the inflamed joint, enhancing their regenerative capacity and therapeutic potential for cartilage repair (172). MSCs are multipotent stromal cells capable of differentiating into various cell types, including chondrocytes, which are essential for cartilage repair (173). In the context of OA, MSCs offer promising therapeutic potential due to their ability to regenerate damaged articular cartilage and modulate inflammation (174). However, their *in vitro* chondrogenic differentiation is slow and often incomplete. Heat shock protein 70, a molecular chaperone induced by mild thermal stress, has emerged as a key enhancer. Periodic heat shock (e.g., 41°C for 1 hour weekly) has been shown to upregulate HSP70 in MSCs, which in turn promotes earlier and more robust chondrogenic differentiation by enhancing the synthesis of cartilage-specific matrix proteins such as type II collagen and aggrecan. Additionally, HSP70 helps prevent chondrocyte apoptosis and may modulate the transition toward hypertrophic differentiation, thereby offering a non-invasive strategy to improve MSC-mediated cartilage regeneration in OA treatment (175).

### 4.7 Ultrasound therapy

Ultrasound (US) therapy is a non-invasive physical treatment modality that uses high-frequency sound waves to penetrate tissues and exert therapeutic effects, particularly in musculoskeletal disorders like knee osteoarthritis (KOA) (176). In the context of OA, US therapy helps alleviate joint symptoms by reducing synovial inflammation, inhibiting chondrocyte apoptosis, and slowing cartilage degeneration (177). US treatment significantly alters the rabbit KOA model’s synovial fluid (SF) proteome, particularly upregulating heat shock proteins such as HSP90A. HSPs, especially HSP90, play a protective role in OA by stabilizing key signaling molecules, blocking apoptosis (e.g., via Apaf-1 inhibition), and enhancing anti-inflammatory pathways such as PI3K-Akt and NF-κB. By increasing HSP expression in SF, US therapy contributes to cellular homeostasis, synovial protection, and potentially cartilage repair, thereby offering a molecular mechanism for its clinical benefits in managing KOA (178).

### 4.8 Pelotherapy

Pelotherapy is a therapeutic practice that uses peloids, natural muds, or clay-like materials for medicinal purposes. These substances are often derived from mineral-rich waters and contain various organic and inorganic compounds, microorganisms, and minerals (179). Chaperokines are members of the molecular HSP families which, when released or exposed extracellularly (e.g., via exosomes, on the cell surface or in free form), function as cytokine- or alarmin-like signaling molecules. They thereby bridge proteostasis (folding, stabilization, degradation) and immune/stress signaling by interacting with extracellular receptors (e.g., TLRs, CD91/LRP1) and modulating innate and adaptive responses (180). Pelotherapy is applied externally (by brush and in mud baths at 38–42°C) for consecutive days. The thermal and chemical stimuli trigger a heat-shock response: intracellular Hsp72 synthesis is promoted while pathological elevations of extracellular Hsp72 (eHsp72), which otherwise act as “chaperokines” driving pro-inflammatory cytokine release, are normalized. By lowering systemic eHsp72, pelotherapy dampens the Hsp-cytokine positive feedback loop and rebalances the neuroendocrine-immune network with a heat-induced boost in cortisol. This anti-inflammatory and chondroprotective mechanism underlies the clinical improvements in pain, stiffness, and function observed in knee OA patients (181).

### 4.9 Electrical stimulation

Mild electrical stimulation (MES) is a noninvasive physical therapy that uses low-intensity direct electrical current to stimulate biological responses in tissues (182). In the OA context, MES has been shown to increase HSP70 levels, a molecular chaperone that helps protect cells from stress and promotes tissue repair. MES does not enhance HSP70 production by boosting gene expression but by inhibiting its degradation via the ubiquitin-proteasome pathway, leading to protein accumulation. When combined with heat stimulation (HS), which induces HSP70 transcription, MES and HS synergistically elevate HSP70 levels more effectively than either treatment alone. This combined treatment enhances cartilage matrix metabolism and upregulates protective proteins like proteoglycans, contributing to chondroprotection and potentially slowing OA progression (183).

### 4.10 Natural products

Natural products play a crucial role in osteoarthritis therapy by providing multi-targeted anti-inflammatory, antioxidant, and chondroprotective effects with fewer side effects than conventional drugs (184). Tunicamycin (TM) is a natural antibiotic that disrupts protein maturation by inhibiting N-linked glycosylation, thereby inducing ERS (185). In OA, TM has been used to model ER stress in chondrocytes, the sole cell type in cartilage responsible for maintaining ECM homeostasis. TM-induced ER stress activates the UPR, initially promoting protective autophagy via the upregulation of GRP78. This chaperone senses misfolded proteins and regulates UPR activation. GRP78-mediated autophagy, marked by increased expression of LC3B and Beclin-1 and autophagosome formation, helps protect chondrocytes from apoptosis. However, persistent or excessive ER stress overwhelms this protective mechanism, suppresses autophagy, and increases cell death. Knockdown of GRP78 significantly reduced autophagy-related protein expression and autophagosome formation, confirming its central role in mediating autophagy during ER stress (186). Melatonin, a natural pineal gland hormone, exerts potent antioxidant, anti-inflammatory, and mitochondrial-protective effects (187). Melatonin alleviates disease progression in OA by inhibiting ferroptosis, an iron-dependent cell death induced by lipid peroxidation, in chondrocytes (188). A key mechanism involves its regulation of NOX4, a ROS-generating enzyme that is upregulated in OA and promotes ferroptosis (189). On one hand, melatonin suppresses NOX4 expression, especially in mitochondria, thus reducing oxidative stress, preserving mitochondrial function, and enhancing energy production (190). Importantly, NOX4 was found to bind directly to GRP78, inhibiting the enzyme GPX4. By inhibiting NOX4, melatonin helps maintain GRP78 function, stabilizes GPX4, and ultimately prevents chondrocyte ferroptosis (189). Conversely, melatonin stabilizes GRP78, easing ER stress, limiting CHOP-driven apoptosis, and reducing misfolded protein buildup in chondrocytes. This stabilization supports chondrocyte survival, reduces cartilage degradation, and ultimately slows OA progression (191). Icariin is a bioactive flavonol glycoside derived from traditional Chinese herbal medicine, widely recognized for its anti-inflammatory, immunomodulatory, cartilage-regenerative, and analgesic effects in OA treatment (192). Icariin targets HSPs, especially HSP90AA1 and HSPA1A, key regulators of protein folding, stress response, and inflammation. By binding HSP90AA1, it influences client proteins controlling chondrocyte survival and inflammatory signaling (MAPK, PI3K/Akt), thereby reducing inflammation, preventing apoptosis, and supporting cartilage homeostasis (193). Icariin dose-dependently inhibits OA–FLS proliferation and migration without cytotoxicity up to 10 μM. It downregulates IL-1β, MMP14, and GRP78 at mRNA and protein levels, thereby reducing inflammation, suppressing ECM degradation, limiting ER stress–mediated apoptosis, and alleviating synovial hyperplasia (194). Similarly, Diacerein, an anti-inflammatory anthraquinone used in OA, acts mainly by inhibiting IL-1β and downstream mediators like NF-κB, thus limiting inflammation and cartilage degradation (195). Diacerein exerts its protective effects in OA through modulation of HSPs, particularly HSP70, which are molecular chaperones that protect cells from stress-induced damage (196). HSP70 can inhibit key inflammatory pathways, suppress chondrocyte apoptosis, and reduce oxidative stress, contributing to cartilage preservation (197). Thus, Diacerein’s therapeutic benefits in OA involve cytokine suppression and enhancement of cellular stress defense mechanisms via HSP regulation (196). Chrysin (CHR) is a natural flavonoid derived from plants such as Scutellaria baicalensis and Mucuna pruriens, known for its anti-inflammatory, antioxidative, and antiapoptotic properties (198) (199). In KOA, CHR has demonstrated therapeutic potential by targeting synovitis and synovial fibrosis, key pathological features of KOA. CHR achieves these effects by modulating the PERK/TXNIP/NLRP3 signaling axis, which is correlated with ERS activity and inflammasome activation in synovial fibroblasts (SFs). Notably, CHR downregulates GRP78, thereby inhibiting downstream activation of PERK and its pro-fibrotic mediators, including CHOP, TXNIP, and NLRP3. This cascade ultimately reduces the production of fibrotic proteins like COL1A1, PLOD2, and TIMP1, and inflammatory cytokines such as IL-6, TNF-α, and IL-1β, alleviating joint inflammation, fibrosis, and pain (199). Taraxasterol (TAX), a pentacyclic triterpene from Taraxacum officinale (dandelion), has long been used in Chinese medicine for its anti-inflammatory properties (200). In OA, TAX reduces joint inflammation and damage by inhibiting TNF-α, IL-6, IL-1β, and NF-κB signaling. It also upregulates miR-140 and miR-146a, which suppress overexpressed targets in OA, including HSPA4L, ST5, and ERBB4, thereby limiting inflammatory responses (201).

Daphnoretin, a natural dicoumarin from plants like Leguminosae and Glycosmis pentaphylla, is known for anti-tumor and antiviral effects. In OA, it promotes chondrocyte survival and inhibits apoptosis by attenuating ER stress and inactivating the NLRP3 inflammasome. Through downregulating ER stress markers (GRP78, CHOP, ATF6, Caspase-12), daphnoretin reduces inflammation, limits apoptosis, and helps preserve joint integrity (202).

Tetramethylpyrazine (TMP), ligustrazine, is a nitrogen-containing compound derived from the Chinese herb Ligusticum wallichii (203). Clinically known for vascular and anti-inflammatory effects, TMP has emerged as a potential OA therapy. It protects chondrocytes by reducing ER stress, primarily through downregulating GRP78 and CHOP, thereby limiting apoptosis. TMP also suppresses cytokines and matrix-degrading enzymes (MMPs, ADAMTS), preserving ECM integrity. Thus, it may alleviate OA by modulating HSP-related ER stress, reducing inflammation, and protecting cartilage (204). Sirtuins (SIRT1–7) play protective roles in osteoarthritis by regulating chondrocyte energy metabolism, oxidative stress, inflammation, autophagy, senescence, and circadian rhythm, thereby maintaining cartilage homeostasis and slowing OA progression (205, 206). Echinacoside (ECH) is a natural phenylethanoid glycoside predominantly found in Cistanche species, widely recognized for its antioxidant, anti-inflammatory, and anti-aging properties (207). ECH protects chondrocytes by targeting oxidative stress and ER stress. It upregulates Sirt1, which alleviates ER stress via the PERK–eIF2α–ATF4–CHOP pathway. By downregulating GRP78 and CHOP, ECH prevents apoptosis and preserves ECM integrity by increasing Collagen II and Aggrecan while reducing MMP13 and ADAMTS5. These effects disappear with Sirt1 silencing or ER stress induction, confirming that ECH mitigates OA mainly through Sirt1-mediated inhibition of HSP-related ER stress (208).

Celastrol, a bioactive compound from Tripterygium wilfordii, shows anti-inflammatory, antioxidant, and anti-apoptotic effects in OA. It protects chondrocytes by inhibiting ER stress via the Atf6/Chop pathway, downregulating markers (Bip, Atf6, Chop, Xbp-1) and reducing apoptotic proteins such as caspase-3 and caspase-9 (209).

Quercetin is a polyhydroxy flavonoid widely present in plants and traditional Chinese herbs, known for its diverse biological activities, including antioxidant, anti-inflammatory, and anti-arthritic effects (210). Quercetin targets key proteins in OA, notably HSPA2 and HSP90AA1, which regulate stress responses and protein homeostasis. By modulating these HSPs, it affects nitric oxide production, estradiol response, and mitochondrial ATP synthesis, processes linked to OA progression. Network pharmacology and docking studies confirmed HSPA2 as a direct target, indicating that quercetin’s protective effects may stem from regulating HSP-mediated stress responses and inflammation, thereby preserving cartilage integrity (211).

Curcumin is a naturally occurring polyphenolic compound with strong antioxidant, anti-inflammatory, and antiapoptotic properties, traditionally used in herbal medicine and as a dietary spice (212). Curcumin protects chondrocytes from oxidative stress–induced apoptosis in OA by upregulating SIRT1, which suppresses the PERK–eIF2α–ATF4–CHOP axis of the UPR. By lowering ER stress markers (CHOP, GRP78, ATF4) and related HSPs, it limits apoptosis, preserves cartilage integrity, and slows OA progression (213).

Baicalin, a flavonoid from Scutellaria baicalensis roots, is noted for its, cytoprotective, antioxidant, and anti-inflammatory effects (214). Baicalin demonstrated protective effects on human chondrocytes, the sole cellular component of articular cartilage, by reducing oxidative stress and preventing apoptosis (215). Its mechanism involves attenuating endoplasmic reticulum stress, contributing to chondrocyte death in OA progression. Specifically, baicalin downregulates ER stress markers such as BiP and CHOP, which are upregulated during cellular stress and promote apoptosis. By suppressing these markers, baicalin helps maintain cellular homeostasis and cartilage integrity, suggesting that modulation of HSP-related ER stress pathways is a central mechanism in its anti-OA activity (216).

Taurine is a naturally occurring, abundant free amino acid in humans, recognized for its roles in calcium modulation, osmoregulation, membrane stabilization, and especially for its antioxidant and anti-apoptotic properties (217). Taurine alleviates ER stress in OA by downregulating HSP-related markers (GRP78, CHOP, Caspase-12) in H_2_O_2_-stimulated chondrocytes. This suppression reduces apoptosis and restores Collagen II production, thereby preserving chondrocyte viability and cartilage function (218).

Harpagide (HPG), an iridoid glycoside from Harpagophytum procumbens, shows strong anti-inflammatory and chondroprotective effects. It suppresses TNF-α–induced inflammation by downregulating IL-1β, IL-6, COX-2, and MMP-13, while restoring COL2A1 and ACAN. HPG also alleviates ER stress by inhibiting GRP78 and p-IRE1α and restoring p-AMPK, suggesting protection via the ER stress/AMPK axis. Through these mechanisms, HPG preserves chondrocyte viability, reduces cartilage degeneration, and improves OA outcomes *in vitro* and *in vivo* (219).

Bushen Zhuangjin decoction (BZD) is a traditional Chinese natural formula composed of ten medicinal herbs, historically used to nourish the liver and kidneys while strengthening tendons and bones (220). BZD has shown therapeutic potential in OA by inhibiting chondrocyte apoptosis, a key factor in cartilage degradation (221). BZD protects chondrocytes from tunicamycin-induced apoptosis by modulating ER stress pathways. It downregulates Bip, Atf4, Chop, Bax, caspase-3, and caspase-9 while upregulating Xbp1 and Bcl-2, thereby limiting ER stress–mediated apoptosis and preserving chondrocyte viability, highlighting its potential as a disease-modifying OA therapy (222).

Similarly, Duhuo Jisheng decoction (DHJSD) is a traditional Chinese herbal medicine composed of multiple botanicals, known for its effects in alleviating OA symptoms by reducing pain, joint stiffness, and improving mobility (223). In OA, chondrocyte apoptosis contributes to cartilage degeneration, often driven by ER stress (6). DHJSD inhibits ER stress–induced apoptosis in chondrocytes, particularly in tunicamycin models, by downregulating miR-34a. This reduces ER stress markers (Bip, Atf4, Chop) and apoptotic proteins (Bax, caspase-9, caspase-3) while upregulating protective factors Xbp1, Xbp1s, and Bcl-2 (224).

## 5 Conclusion

Osteoarthritis is not merely a problem of “wear and tear,” but a stress-response disease in which proteostasis failure, ER stress, and chaperone biology decisively shape chondrocyte fate, extracellular matrix turnover, and synovial inflammation. Across the evidence reviewed here, HSPs, notably HSP27, HSP40, HSP60, HSP70, HSP90, and the ER sentinel GRP78/BiP, form an integrated control layer that determines whether stressed joint cells adapt and survive or enter catabolic, apoptotic, and fibrotic programs. This review highlights a consistent theme: the same chaperone can be protective in one context (restoring folding, suppressing apoptosis, enabling autophagy) yet pathogenic in another (amplifying DAMP/TLR signaling, sustaining NF-κB activation, or stabilizing pro-catabolic client proteins). Appreciating this compartment- and context-specific duality is central to designing disease-modifying strategies.

Among the chaperones, HSP70 stands out for its location-dependent effects. Intracellular HSP70 (iHSP70) is broadly cytoprotective, limiting caspase activation, buffering oxidative stress, supporting mitochondrial function, and preserving ECM synthesis, whereas extracellular HSP70 (eHSP70) acts as an alarmin that can drive TLR-NF-κB inflammation in synovium. Therapeutic approaches that increase iHSP70 (e.g., controlled thermal stimulation, certain small molecules, metabolic adjuvants) while avoiding eHSP70 spillover offer a rational way to tip the balance toward repair. Transient GRP78-driven UPR helps clear misfolded proteins and restores homeostasis; persistent activation under mechanical overload, hypoxia, high glucose, or pollutant exposure switches signaling toward CHOP/caspase-12 apoptosis, proliferative arrest, ferroptosis susceptibility, and ECM degradation. This adaptive-to-maladaptive transition explains why ER-stress modulators can be protective only within a defined “dose–time” window.

GRP78 induction in osteoarthritis appears to exert dual and stage-dependent effects rather than being purely protective or pathogenic. In the early phase of OA or immediately following mechanical or metabolic stress, moderate GRP78 upregulation reflects an adaptive unfolded-protein response that temporarily enhances proteostasis, limits ER overload, and supports chondrocyte survival. However, as stress becomes chronic or overwhelming—through sustained mechanical loading, hypoxia, inflammation, or glycation—persistent GRP78 activation sustains unfolded-protein-response signaling, promotes CHOP- and caspase-12–mediated apoptosis, and amplifies matrix degradation. Thus, GRP78 functions as a molecular rheostat: its transient activation is cytoprotective, but prolonged elevation signals maladaptation, driving chondrocyte death and ECM breakdown. Clinically, its progressive increase in synovial fluid with advancing K–L grades suggests GRP78 may serve as a biomarker of ER stress burden and disease progression, while its modulation could represent a stage-specific therapeutic target—enhancing its early adaptive capacity but restraining its late pro-apoptotic output.

HSP90 illustrates the flip side of chaperone biology in OA. By stabilizing numerous client proteins, including kinases and transcription factors, HSP90 can sustain NF-κB signaling, promote nitric-oxide-mediated chondrocyte injury, and, through its mitochondrial isoform TRAP1, reinforce TGF-β/Smad-driven synovial fibrosis. Necroptotic release of HSP90 also functions as a DAMP that perpetuates inflammation. Importantly, selective HSP90 inhibition not only diminishes these catabolic cues but also disinhibits HSF1, secondarily boosting protective HSP70, an example of “network-aware” intervention that realigns the stress proteome toward resilience. HSP27 and HSP40/DNAJ cofactors fine-tune the HSP70 cycle and inflammatory output: MK2-phosphorylated HSP27 couples cytokine stimulation to PGE2 and MMPs, yet HSP27 is also leveraged by TSP-1 to enhance chondrocyte autophagy and survival. HSP60, though often discussed in autoimmunity, supports chondrocyte mitochondrial integrity and stabilizes SOX9; its deficiency in damaged cartilage aligns with impaired anabolic capacity and heightened inflammatory tone.

As summarized in **Table 2**, HSPs in OA do not behave as uniformly “good” or “bad” actors but as highly context-dependent regulators. HSP40 and HSP60 are predominantly cytoprotective, whereas HSP27, HSP70, GRP78 and HSP90 function as double-edged swords whose net effect depends on localization (intra- vs extracellular), intensity and duration of stress (mechanical, metabolic, hypoxic, ER), and post-translational regulation. These nuances argue that future HSP-targeted therapies must aim to restore physiological, compartment-specific chaperone activity rather than simply inhibiting or globally boosting a given HSP.

**Table 2:** Context-dependent protective versus pathogenic roles of major HSP families in osteoarthritis, indicating whether each acts predominantly protective, predominantly harmful, or as a double-edged sword depending on the stimulus and cellular compartment.

HSP family	Protective effect	Pathological effect
HSP27	Mainly protective: during development and in OA under adaptive/repair stress (e.g. TSP-1–induced), promotes autophagy and chondrocyte/ECM homeostasis.	Double-edged: under IL-1β/TNF-α with p38–MK2 activation, phosphorylated HSP27 drives PGE2 and MMP3/13 → inflammation and cartilage loss.
HSP40 (DNAJB12)	Protective in physiological conditions: normal DNAJB12 supports ER-associated autophagy, clears misfolded proteins, and limits ER stress.	No clear pathogenic role in normal OA; mainly implicated as a genetic risk factor when altered/dysregulated.
HSP60	Predominantly protective: adequate HSP60 (e.g. estradiol-induced) supports mitochondria, stabilizes SOX9, boosts collagen II/aggrecan, and reduces IL-1β/VEGF and synovial inflammation.	No established direct pathogenic effect in OA; levels are reduced in OA, suggesting loss of protection rather than active harm.
HSP70 (HSP72, HSPA1A, HSPA5)	Protective intracellularly: iHSP70/HSPA1A/HSP72/HSPA5 up in adaptive stress (heat, exercise, GGA, moderate loading, OS) → proteostasis, anti-apoptotic, anti-inflammatory, anti-ferroptotic, and ECM-preserving.	Double-edged: eHSP70 and stress-overactivated HSP70 (obesity/insulin resistance, excessive load) signal via TLRs/NF-κB, ↑MMPs and inflammation; levels rise with KL grade and can promote degeneration.
GRP78 (BiP, HSPA5)	Protective in early/moderate ER stress: adaptive UPR (IRE1–XBP1, transient PI3K/Akt, mTOR suppression) enhances autophagy and helps chondrocyte survival, especially at OA onset.	Double-edged: with chronic/intense ER stress (mechanical overload, hypoxia, high glucose, AGEs, toxins), GRP78-driven UPR shifts to PERK–CHOP/caspase-12, Skp2↓/p27↑, ↑MMPs/ADAMTS → apoptosis and ECM loss.
HSP90 (HSP90β, TRAP1)	Context-limited protection: TRAP1 up in hypoxic deep cartilage supports mitochondrial/stress adaptation; HSP90β can repress MMP-13 in some low-OA phenotypes.	Mainly pathogenic: acts as DAMP from necroptotic chondrocytes, promotes NF-κB activation, NO-mediated death, MMP-13 and synovial fibrosis (TRAP1/TGF-β/Smad). Overall HSP90 activity in OA is pro-degenerative; its inhibition is chondroprotective.

These mechanistic insights translate into a diversified, yet increasingly coherent, therapeutic landscape. Proteostasis-oriented small molecules (e.g., client-selective HSP90 inhibitors such as ITZ-1, proteasome-linked HSP70 induction with MG132, or COX-2–centric agents that also down-shift HSP90/HSP70 expression) dampen inflammatory and degradative loops while sparing essential housekeeping. Bioactives from natural products (curcumin, baicalin, taurine, vitexin, harpagide, and others) converge on ER-stress axes and redox control, frequently normalizing GRP78/HSP70 dynamics, curbing MMPs and COX-2, and restoring COL2A1/ACAN expression in preclinical models. Physical modalities, near-infrared photothermy (e.g., ICG-mediated), microwave deep heating, and controlled hyperthermia, offer non-pharmacologic induction of iHSP70 with measurable gains in matrix metabolism when dosed within safe thermal envelopes. At the delivery frontier, nanomedicines (gold-nanocage systems, nanozymes) couple on-demand drug/siRNA release with photothermal HSP70 induction and ROS scavenging; extracellular vesicles and mesenchymal-stem-cell–based approaches exploit HSP-enriched paracrine cargo and stress-resilience programming to promote cartilage repair and synovial immunomodulation. First-in-class biologics (e.g., anti-HSP90α antibodies) point to isoform-specific, pathway-aware targeting capable of deflating glycolytic and inflammatory drivers in hypoxic cartilage.

A growing body of preclinical data suggests that locally delivered, HSP-modulating strategies with some existing clinical track record are the most promising routes toward translation, particularly intra-articular small molecules (e.g., selective HSP90 modulators, ER-stress inhibitors), nanocarriers and EV-based therapies, and physical modalities that boost protective HSP70 without systemic exposure. Among these, HSP-selective small molecules and biologics (e.g., client-selective HSP90 inhibitors, 9B8 mAb) are attractive because their pharmacology can be finely tuned and monitored, but their success will ultimately depend on achieving joint-restricted exposure to avoid off-target toxicity on ubiquitous HSP networks (e.g., cardiotoxicity, impaired stress tolerance, tumor surveillance). Nanomedicine and MSC/ACSC-derived EVs are particularly compelling as “precision” delivery systems to chondrocytes and synoviocytes and as endogenous HSP70/GRP78 carriers, yet they face major hurdles including rapid synovial clearance, immunogenicity, batch-to-batch heterogeneity, and complex CMC/regulatory pathways. Physical and thermal modalities (microwaves, laser, MES + heat, pelotherapy) are conceptually appealing, low-cost adjuncts that upregulate intra-articular HSP70 while avoiding pharmacologic off-target effects, but require standardization of dosing (intensity, duration, frequency) and rigorous placebo-controlled trials. Finally, although natural products and multi-herb formulas provide rich, multitarget HSP and ER-stress modulation, their translation is limited by poor bioavailability, pleiotropic targets, and difficulties in defining pharmacodynamic windows. Overall, the most realistic near-term candidates are locally administered agents (small molecules, nanoformulations, EVs) that selectively enhance cytoprotective HSP responses in cartilage and synovium while minimizing systemic exposure, supported by robust delivery strategies, biomarker-guided dosing, and careful assessment of long-term off-target and immunological effects. As demonstrated in **Supplementary Table 1**, the regulatory effects of these treatments appear to be largely non-selective, suggesting that they may modulate not only HSP expression but also a broader range of molecular targets involved in osteoarthritis pathophysiology. This pleiotropic activity underscores the complexity of interpreting their outcomes and highlights the need for strategies that more precisely delineate HSP-specific effects from off-target mechanisms.

For translation, three principles emerge. First, phenotype and stage matter: early OA marked by adaptive UPR imbalance may benefit most from ER-stress buffering and iHSP70 up-regulation; obesity/metabolic OA might require strategies that reduce eHSP70-TLR signaling and restore insulin-linked HSP70 expression; fibrotic, pain-dominant phenotypes could need TRAP1/HSP90-axis interference alongside anti-fibrotics. Second, compartment and delivery matter: maximizing intra-articular exposure while minimizing systemic off-target effects (especially for HSP90 inhibitors) is essential; thermal or light-triggered, joint-confined approaches are attractive for this reason. Third, biomarkers and response monitoring are feasible: synovial-fluid and plasma HSP70/GRP78 levels, ECM turnover markers, and imaging-integrated readouts can index disease activity and therapeutic engagement. Building composite panels that capture both proteostasis and inflammation should improve patient selection and endpoint sensitivity.

We therefore propose a pragmatic, staged framework for clinical development:

1. Restore adaptive proteostasis early (ER-stress modulators, controlled iHSP70 induction, antioxidant/mitochondrial support);
2. Interrupt catabolic nodes mid-course (HSP90/TRAP1 blockade, MK2–HSP27 axis modulation, selective client degradation), ideally paired with graded heat/photothermal sessions to sustain iHSP70;
3. Rebuild and defibrose late (MSC/EV therapies tuned for HSP cargo, anti-fibrotic targeting of synovium, nano-enabled delivery of chondrogenic cues), all layered atop guideline care (weight loss, strength training, biomechanical correction, analgesic stewardship).

Toward phenotype-guided translation, the proposed staged framework should not be applied uniformly but stratified according to OA endotypes. Patients with metabolic OA, characterized by obesity, insulin resistance, and low-grade inflammation, often display elevated extracellular HSP70 and GRP78 in plasma and synovial fluid, suggesting that therapies dampening eHSP70-TLR signaling and restoring insulin-linked iHSP70 expression may be most effective. In contrast, post-traumatic or mechanically driven OA with focal cartilage injury may respond better to localized proteostasis restoration (controlled thermal iHSP70 induction, ER-stress modulators) in early phases. Fibrotic or pain-dominant phenotypes marked by persistent synovial TRAP1/HSP90 activation could benefit from anti-fibrotic and HSP90-targeted strategies in later stages. Integrating biomarker panels, including synovial-fluid or plasma HSP70/GRP78 levels, matrix turnover indices (COMP, CTX-II), and imaging readouts, can enable patient stratification, stage-specific enrollment, and pharmacodynamic monitoring, aligning HSP-targeted approaches with personalized-medicine principles.

The promise of targeting HSPs is tempered by context dependence: the same chaperone can be cytoprotective intracellularly yet pro-inflammatory when extracellular, and many preclinical systems fail to capture this compartment specificity. Common *in vitro* (IL-1β/TNF-α, high-glucose, mechanical overload) and acute rodent models incompletely mirror human, slowly progressive, multi-tissue OA, so doses and timings that rebalance the UPR adaptively in animals may drive maladaptive stress in patients. Heterogeneity in assays (isoform- and compartment-specific HSP measurements), outcome definitions, and imaging/biomarker panels limits cross-study comparability, while long-term safety of chronic HSP modulation, repeated thermal/photothermal exposure, and sustained HSP90 inhibition remains insufficiently characterized with respect to host defense, tumor surveillance, and tissue remodeling. Evidence maturity also varies across modalities (small molecules vs. biologics vs. nanomedicine vs. cell/EV therapies), creating a translation gap with few head-to-head, dose-finding, or phenotype-stratified clinical trials. As a result, mechanistic attribution can be uncertain—many agents act pleiotropically, so linking benefit specifically to HSP/ER-stress axes is challenging when controls are incomplete and publication bias favors positive preclinical signals.

Collectively, targeting HSPs offers a unifying pathobiological lens and a tractable therapeutic handle for OA. By moving from single-target symptom control toward network-aware, context-specific proteostasis therapy, it should be possible to preserve viable chondrocytes, stabilize matrix biology, temper synovitis and fibrosis, and ultimately change the slope of disease. Realizing this promise will require rigorously phenotyped trials, standardized thermal/dosing protocols, compartment-specific delivery systems, and safety monitoring attuned to chaperone network effects. With these elements in place, HSP-targeted interventions can plausibly transition from compelling preclinical signals to genuine disease-modifying therapies for patients with OA.
