# Supplementary material for: Heat shock proteins in osteoarthritis: molecular mechanisms, pathogenic roles, and therapeutic opportunities
Source: Front Immunol. 2025 Dec 15;16:1688250. doi: 10.3389/fimmu.2025.1688250 (PMC12745223; doi:10.3389/fimmu.2025.1688250)
Supplement: Supplementary file 1 [file Table1.docx]

| Name of Treatment  **Supplementary Table 1**-Different treatments targeting HSP proteins in OA. | Treatment Category | Chemical Structure | Targeted HSP Protein(s) | Other targeted Protein(s) | Signaling pathway(s) Affected | Model of Experiment | Study Key Findings | Ref. |
| --- | --- | --- | --- | --- | --- | --- | --- | --- |
|  | **Synthetic Products** | | | | | | | |
| HMW-HA | High-molecular-weight HA | 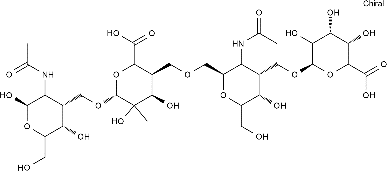 | ↓HSP70 (GRP78) | IL-6, IL-8, TNF-α, IL-10, iNOS, COX2, PGE-S | GRP78–NF-κB signaling | IL-1β-treated OA synoviocytes,THP  -1 macrophages | Suppresses GRP78, reduces NF-κB activation, lowers IL -6/PGE2, promotes M2 macrophage polarization | [144] |
| VA692 | Selective COX-2 inhibitor + NO donor | 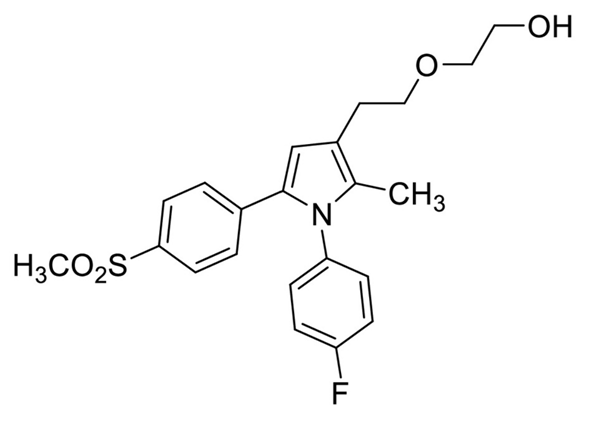 | ↓HSP90α, HSP90β, HSP70 (HSP7C), HSP10 | COX-2, IL-1β, IL-6, IL-8, SOD-2, CAT, SOD1 | Inflammation, Oxidative Stress, Apoptosis, Cytoskeletal remodeling | IL-1β-stimulated human chondrocyte cell line (T/C-28a2), Primary human OA chondrocytes | Downregulates HSPs involved in OA pathology, reduces ROS, inflammation, and apoptosis, modulates calcium signaling, and restores cytoskeletal integrity. | [145] |
| ITZ-1 | Small molecule; client-selective Hsp90 inhibitor | 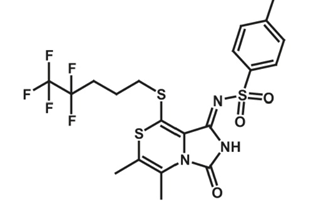 | ↑Hsp90 (C-terminal binding) | Raf-1, HSF1 | ERK/MAPK signaling, HSF1 activation | Human articular chondrocytes (HACs), in vitro and in vivo OA models | Inhibits IL-1β–induced MMP-13 via Raf-1 degradation; induces Hsp70 expression through HSF1 activation; minimal cytotoxicity; promising disease-modifying anti-OA agent | [146] |
| MG132 | Proteasome inhibitor | 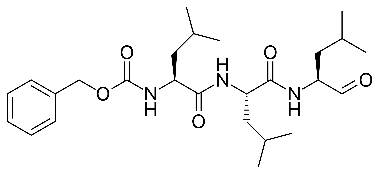 | ↑Hsp70 | Caspase-3, NF-κB targets | Ubiquitin–proteasome, NF-κB, ER stress | ACLT-induced rat OA | Induced Hsp70 expression; reduced chondrocyte apoptosis; decreased synovitis; anti-inflammatory | [148] |
| 4-PBA | ER stress inhibitor | 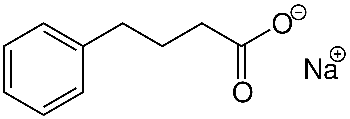 | ↓HSP70 (GRP78) | CHOP, Bcl-2, Bax, Caspase-3, MMP2, MMP9, FAP | ER stress, UPR, mitochondrial apoptosis | Rat OA model (ACLT-induced) | 4-PBA inhibits ER stress via downregulating GRP78 and CHOP, suppresses apoptosis and inflammation, and protects cartilage from OA-induced damage. | [150] |
|  | **Nanoparticles** | | | | | | | |
| AuDPNAs + Laser | Nanocarrier (AuNCs + DIA + siNGF) | - | ↑HSP-70 | NGF, COL2A1, ACAN, MMP-13, ADAMTS-5, TNF-α, IL-6 | Photothermal + ROS inhibition | DMM-induced OA in mice | Light-triggered DIA/siNGF release; enhances chondrocyte survival; reduces inflammation and oxidative stress; upregulates HSP-70; improves joint function. | [153] |
| MPMP nanozyme + NIR | Nanozyme / PTT agent | - | ↑HSP70 | IL-6, TNF-α, MMP-13, ACAN, COL2A1, HAS2 | NF-κB, IL-17, MAPK, HSP-related signaling | MIA-induced OA mice | Mimics antioxidases and HAS; upregulates HSP70; scavenges ROS/RNS; promotes chondrogenesis and HA synthesis; restores joint homeostasis. | [154] |
| MPMP nanozyme (no NIR) | Nanozyme | - | ↑HSP70 | IL-6, TNF-α, MMP-13 | NF-κB suppression | In vitro + OA mice | Antioxidase-mimicking; partial anti-inflammatory effect; limited chondrogenesis without photothermal activation. | [154] |
|  | **Extracellular Vesicles** | | | | | | | |
| CPC-derived EVs | Cell-free biologic | - | HSP70 | CD9, CD63, CD81 | Paracrine/Anabolic | In vitro human fibrochondrocytes | Improved viability, migration, COL2A1 upregulation; optimal at 10⁷ particles/mL | [159] |
| BM-MSC-derived EVs | Cell-free biologic | - | HSP70 | CD9, CD63, CD81 | Paracrine signaling | In vitro human fibrochondrocytes | Modest increase in viability; lower wound healing efficacy than CPC-EVs | [159] |
|  | **Monoclonal antibody** | | | | | | | |
| 9B8 | Monoclonal antibody | 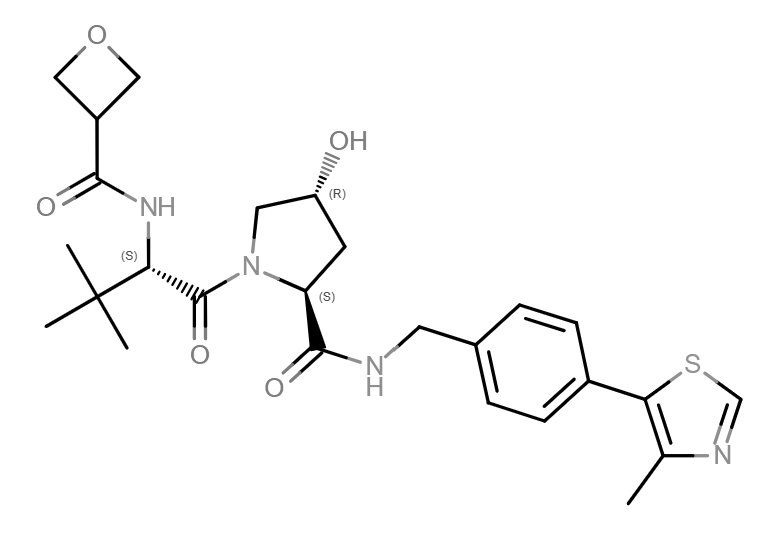 | ↓HSP90α | ENO2, SLC2A1, PFKP, MMP13, ADAMTS4, IL-6, TNF-α | HIF-1α / Glycolysis | In vitro (human chondrocytes), In vivo (ACLT rat) | Suppresses HIF-1α signaling, inhibits glycolysis and inflammation, protects cartilage and bone | [160] |
|  | **Irradiation Therapy** | | | | | | | |
| ICG + NIR | Photosensitizer + NIR | - | ↑HSP70 | ACAN, SOX9 | Photothermal stimulation via NIR | In vitro (rat chondrocytes); In vivo (rat knees) | Safely increased intra-articular temperature to ~40 °C; upregulated HSP70 and ACAN; enhanced cartilage metabolism; no observed tissue damage. | [164] |
| ICG + NIR + Quercetin | Photosensitizer + HSP inhibitor | - | ↓HSP70 (inhibited) | ↓ ACAN, ↓ SOX9 | HSP70 inhibition pathway | In vitro | Quercetin blocked HSP70 expression and reduced ACAN/SOX9 induction, confirming HSP70's mediating role in cartilage metabolism enhancement. | [164] |
| Hyperthermia | Physical therapy | - | ↑HSP70 | Type II collagen, Aggrecan | Stress response, apoptosis | In vitro, a Rabbit model | Increases HSP70 expression; boosts cartilage metabolism; inhibits chondrocyte apoptosis | [165] |
| Microwave therapy | Physical therapy | - | ↑HSP70 | Type II collagen, Aggrecan | Stress response, metabolic boost | Rabbit knee OA model | Induces HSP70 in deep cartilage; improves matrix synthesis; optimal intensity needed for effect | [165] |
| Microwave thermotherapy | Physical therapy | - | ↑HSP70 | Proteoglycan (PG), Col II | Heat-stress response | In vivo (Rabbit knee) | Increased intraarticular temperature (optimal at 40 W) upregulated HSP70; enhanced PG and Col II mRNA expression; HSP70 linked to PG regulation. | [165] |
| Quercetin + Microwave | Physical Therapy Natural Products | - | ↑HSP70 | Proteoglycan (PG) | HSP70 suppression pathway | In vivo (Rabbit knee) | Quercetin inhibited heat-induced HSP70 and PG expression, indicating PG is HSP70-dependent; Col II was unaffected, suggesting alternate regulation. | [165] |
| Microwave irradiation (MW) | Physical/thermal stimulation | - | ↑HSP70 | Aggrecan, Col II | Stress-induced chondroprotection | Rat OA model (ACLT) | MW at 40 W induces moderate intraarticular heat (~38.3°C), upregulates HSP70 and enhances cartilage metabolism | [166] |
| MW + Gln | Combination therapy | - | ↑HSP70 | Aggrecan↑, Col II (NS)↑ | HSP70-mediated ECM protection | Rat OA model | The most effective combination for increasing HSP70, aggrecan, and protecting against OA degeneration | [166] |
| Laser Irradiation | Physical Therapy | - | ↑HSP70 | IL-1β, TNF-α | Anti-inflammatory, chondroprotection | C57BL/6 mice with OA | Promotes HSP70 expression; reduces inflammatory cytokines; alleviates OA symptoms | [167] |
|  | **Stem cell therapy** | | | | | | | |
| ACSCs | Stem cell therapy | - | ↑GRP78 (BiP) | PERK, ATF4, TMEM119, BMP6, CDK4, Cyclin D, Bcl2, Bax, Caspase-3 | PERK–eIF2α–ATF4 (UPR) | Human C28/I2 chondrocyte line | Reduced ERS-induced apoptosis; restored cell cycle and viability via modulation of PERK and HSPs | [171] |
| Pelleted hBMMSCs (Human Bone Marrow MSCs) | Cell-based therapy | - | ↑HSP70 | BAX, BCL2, P53, TNF-α, Cyclin A2/E2 | Heat shock response, apoptosis | In vitro (OA patient cells) | Pelleted MSCs showed greater resistance to arthroscopic thermal stress than cell suspensions. Increased HSP70 in pellets suggests a heat-adaptive, cytoprotective response. Suppressed apoptotic and inflammatory gene expression compared to suspensions. | [172] |
|  | **Physical Therapy** | | | | | | | |
| Periodic Heat Shock | Physical (thermal stimulus) | - | ↑HSP70 | COL2A1, ACAN, COL1A1, COL10A1 | Heat shock response pathway | 3D pellet culture of hMSCs (human) | Accelerates chondrogenic differentiation of hMSCs, increases type II collagen and aggrecan expression, induces HSP70, and promotes early maturation. | [175] |
| Ultrasound (US) | Physical therapy | - | ↑HSP90 (HSP90A) | DCN, PK/PKY, FABP4/aP2, ApoA-I, TF, CBP2, PON, A2M, Fibrinogen α-chain | PI3K-Akt, NF-κB, TGF-β, PPAR, complement and coagulation cascades, glycolysis/gluconeogenesis, HIF-1, estrogen signaling | Rabbit KOA model (ACLT) | US upregulated HSP90A, DCN, PK, and FABP4 while downregulating pro-inflammatory proteins such as ApoA-I, TF, PON, and A2M in the synovial fluid of KOA rabbits | [178] |
|  | Pelotherapy | | | | | | | |
| Mud therapy (pelotherapy) | Non-pharmacological (balneotherapy) | - | ↓Hsp70 (Hsp72/eHsp72) | IL-1β, TNF-α, IL-8, IL-6, TGF-β, Cortisol | Neuroendocrine-immune feedback, HPA axis modulation | Human subjects (OA patients, aged 62–77) | Reduced systemic inflammatory cytokines, increased cortisol, decreased eHsp72; improved pain, function, and quality of life | [181] |
|  | Electrical Stimulation | | | | | | | |
| Mild Electrical Stimulation (MES) | Electrical Stimulation | - | ↑HSP70 | Ubiquitinated proteins | Proteasome inhibition | In vitro (rabbit chondrocytes), In vivo (rat knee) | Increases HSP70 protein by preventing degradation; enhances cartilage matrix metabolism | [183] |
| Heat Stimulation (HS) | Electrical Stimulation | - | ↑HSP70 | PG (Proteoglycan), col2 (Type II collagen) | Transcriptional activation | In vitro, In vivo | Induces HSP70 gene expression and promotes matrix gene transcription | [183] |
| MES + HS | Electrical stimulation plus heat therapy | - | ↑HSP70 | PG, ubiquitinated proteins | Proteasome inhibition + transcriptional activation | In vitro, In vivo | Synergistically increases HSP70 protein; significantly enhances PG expression; a potential safe, noninvasive OA therapy | [183] |
| Microwave heat + glutamine | Electrical stimulation and chemical combination | - | ↑HSP70 | – | Transcriptional activation + metabolic enhancement | In vivo (rat model) | Shown to suppress OA progression via enhanced HSP70 expression | [183] |
|  | **Natural Products** | | | | | | | |
| Tunicamycin | ER stress inducer | 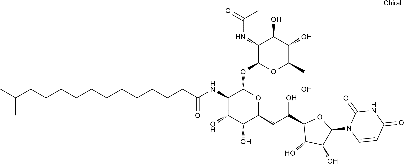 | ↑GRP78 (HSPA5) | ATF4, CHOP, GADD34, LC3B, Beclin-1 | UPR–autophagy–apoptosis axis | Primary rat chondrocytes | TM induces ER stress, activates UPR, and autophagy via GRP78. Early autophagy protects chondrocytes, but prolonged stress leads to apoptosis. | [186] |
| Melatonin | Natural hormone/antioxidant | 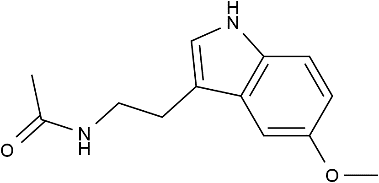 | ↑HSP70 (GRP78/BiP) | NOX4, GPX4, MMP13, COL2A1 | Ferroptosis, oxidative stress, mitochondrial dysfunction | In vitro (chondrocytes), in vivo (ACLT mouse model), and human cartilage samples | Melatonin inhibits ferroptosis in OA by downregulating NOX4, preserving mitochondrial function, enhancing GPX4 via GRP78 stabilization, reducing inflammation, and cartilage degradation. | [189] |
| Melatonin | Endogenous hormone | 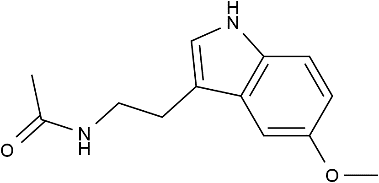 | ↑HSP70, GRP78 (HSPA5) | CHOP, SIRT1, XBP1S, IRE1α, Bcl-2, Bax | IRE1α–XBP1S–CHOP signaling, ER stress/UPR | In vitro (human chondrocytes), in vivo (mouse ACLT model) | Upregulates GRP78, reduces ER stress and apoptosis, activates SIRT1, and inhibits OA progression. | [191] |
| Icariin | Natural flavonol glycoside (herbal compound) | 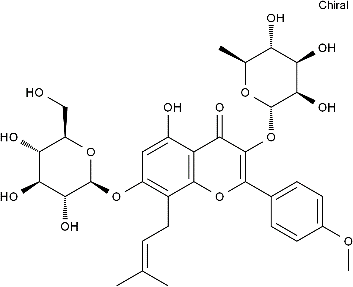 | ↓HSP90AA1, HSPA1A | SRC, MAPK1, AKT1, PTPN11, ESR1, EGFR, RHOA, JAK2, MAPK14 | MAPK, PI3K/Akt, Estrogen, FOXO, Ras signaling | Network pharmacology and molecular docking | Targets HSP90AA1 and HSPA1A to regulate protein folding and inflammation; Modulates MAPK and PI3K/Akt pathways to protect chondrocytes and reduce OA progression | [193] |
| Icariin | Natural flavonoid (from *Epimedium*) | 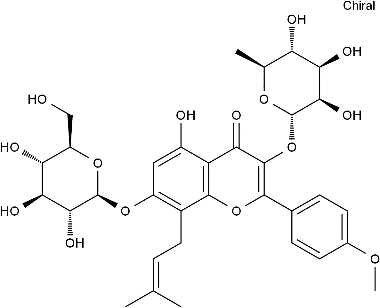 | ↓HSP70 (GRP78) | IL-1β, MMP14 | ER stress, inflammation, ECM degradation | Human OA–FLSs (in vitro) | Inhibits FLS proliferation and migration; downregulates GRP78, MMP14, and IL-1β; reduces ER stress and inflammatory response; potential therapeutic for OA | [194] |
| Diacerein | Anthraquinone anti-inflammatory | 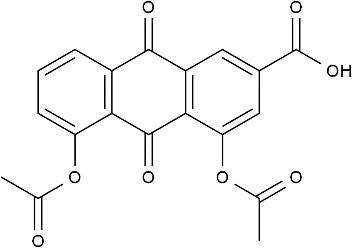 | ↑HSP70 | IL-1β, NF-κB, MMPs | IL-1β/NF-κB signaling, HSP-mediated cytoprotection | OA in vitro/in vivo models | Inhibits IL-1β and NF-κB; reduces inflammation and cartilage degradation; upregulates HSP70 for cytoprotection | [196] |
| Chrysin (CHR) | Flavonoid | 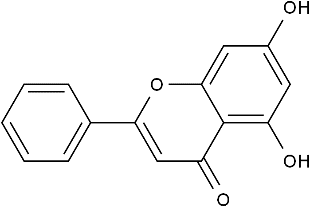 | ↑HSP70 (GRP78) | TGF-β, COL1A1, PLOD2, TIMP1, IL-6, IL-1β, TNF-α, CHOP, TXNIP, NLRP3 | PERK/TXNIP/NLRP3 signaling | In vivo (ACLT-induced KOA rats); In vitro (TGF-β-stimulated SFs) | Reduces synovial inflammation and fibrosis; inhibits ER stress and inflammasome activation via HSP70 pathway | [199] |
| Taraxasterol | Triterpene | 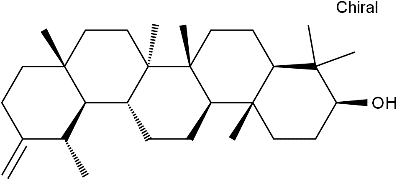 | ↓HSP70 (HSPA4L) | ST5, ERBB4, S100A8, CCL3, A2M, LBP, CCR1 | NF-κB signaling | Papain-induced OA rat model | Suppresses inflammation via NF-κB inhibition; upregulates miR-140 and miR-146a; downregulates HSPA4L and other inflammatory mediators in OA joints. | [201] |
| Daphnoretin | Natural dicoumarin | 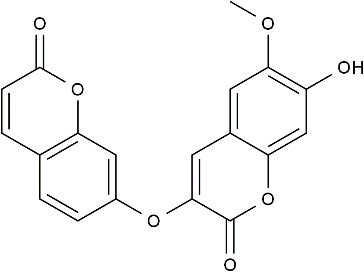 | ↓HSP70 (GRP78) | CHOP, ATF6, Caspase-12, NLRP3, ASC, Caspase-1, COX-2, iNOS, TNF-α, IL-6 | ER stress (ERS), NLRP3 inflammasome | IL-1β-induced chondrocytes (in vitro), DMM mouse model (in vivo) | Daphnoretin alleviates OA by reducing ER stress and NLRP3 inflammasome-mediated inflammation and apoptosis, enhances chondrocyte viability, and preserves cartilage integrity. | [202] |
| Tetramethylpyrazine (TMP) | Natural alkaloid (from Ligusticum wallichii) | 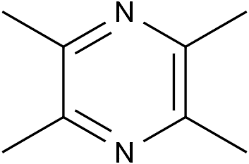 | ↓GRP78 (HSPA5) | CHOP, IL-1β, IL-6, TNF-α, COX-2, iNOS, MMP-3, MMP-13, ADAMTS-4, ADAMTS-5 | ER stress/UPR, NF-κB | In vitro (rat chondrocytes), TG and IL-1β stimulation | Reduces ER stress, inflammation, and apoptosis; preserves ECM integrity; downregulates HSP-related stress signals | [204] |
| Echinacoside | Natural compound | 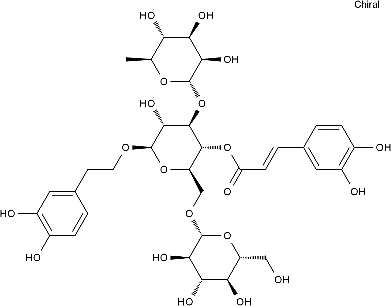 | ↓HSP70 (GRP78) | Sirt1, CHOP, ATF4, PERK, BAX, Bcl-2, eIF2α | PERK–eIF2α–ATF4–CHOP | In vitro (chondrocytes), in vivo (DMM) | Reduces ER stress and apoptosis via Sirt1; preserves ECM integrity; mitigates OA progression. | [208] |
| Celastrol | Natural compound | 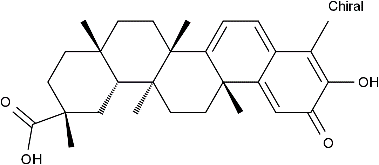 | ↓HSP70 (GRP78) | ATF6, CHOP, XBP1, Caspase-3, -6, -9 | ER stress (Atf6/Chop) | In vitro (Tm-induced chondrocytes); In vivo (ACLT-induced OA rats) | Suppresses ER stress-induced apoptosis; downregulates Bip, ATF6, CHOP, XBP1 and caspases | [211] |
| Quercetin (from Achyranthes bidentata) | Flavonoid | 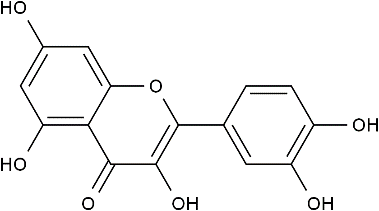 | HSPA2, HSP90AA1 | PIM1, CYP1B1, ESR1, ESR2, ACTB, ATP5A1, ATP5B, ATP5C1, CSNK2A1, CSNK2B, RUVBL2 | Nitric oxide biosynthetic process; Estrogen signaling; Mitochondrial ATP synthesis | Network pharmacology; GSE55457 transcriptome; Molecular docking | Targets HSPs to modulate cellular stress and inflammation; strong docking with OA-related proteins; supports cartilage protection | [213] |
| Curcumin | Natural polyphenol | 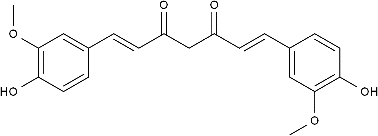 | ↓HSP70 (via GRP78) | SIRT1, CHOP, ATF4, PERK, eIF2α, Bcl2, Caspase3, PARP | PERK-eIF2α-ATF4-CHOP signaling; ER stress and oxidative stress pathways | TBHP-induced chondrocyte injury (in vitro); ACLT rat OA model (in vivo) | Curcumin activates SIRT1, inhibits ER stress, reduces HSP70 (GRP78), and prevents chondrocyte apoptosis to attenuate OA progression. | [215] |
| Baicalin | Flavonoid (Traditional Chinese Medicine) | 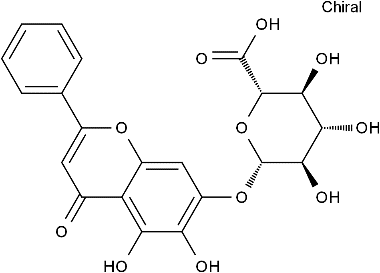 | ↓HSP70 (BiP/GRP78) | CHOP, Bax, Bcl-2, Caspase-3, Collagen I, Collagen II, Aggrecan, Sox9 | ER stress attenuation; apoptosis inhibition; oxidative stress response | Human OA chondrocytes treated with H₂O₂ | Baicalin protects chondrocytes by reducing ER stress and oxidative damage, modulates apoptosis-related genes, and preserves ECM integrity | [218] |
| Taurine | Endogenous amino acid | 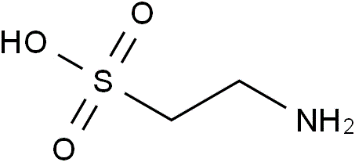 | ↓GRP78 (HSPA5) | GADD153 (CHOP), Caspase-12, Collagen II | ER stress-apoptosis | Human OA patient-derived chondrocytes (in vitro), cartilage samples (ex vivo) | Taurine alleviates H₂O₂-induced ER stress by downregulating HSPs and apoptotic markers while restoring Collagen II synthesis and improving viability. | [220] |
| Bushen Zhuangjin Decoction (BZD) | Traditional Chinese Medicine | Mixed | ↓HSP70 (Bip/GRP78) | Xbp1, Atf4, Chop, Bax, Bcl-2, Caspase-3, Caspase-9 | ER Stress-mediated apoptosis | TM-induced chondrocyte apoptosis (in vitro) | Inhibits chondrocyte apoptosis by suppressing ER stress and modulating HSP70-related UPR pathways | [224] |
| Duhuo Jisheng decoction (DHJSD) | Traditional Chinese Medicine (TCM) | Mixed | ↓HSP70 (GRP78/Bip) | Atf4, Chop, Xbp1, Xbp1s, Bcl-2, Bax, caspase-9, caspase-3, miR-34a | ER stress-mitochondrial apoptotic pathway | Tunicamycin-induced chondrocyte ER stress (in vitro) | DHJSD reduces chondrocyte apoptosis by inhibiting ER stress and downregulating miR-34a, suggesting protective effects via HSP70/Bip modulation. | [226] |
| Harpagide (HPG) | Natural iridoid glycoside | 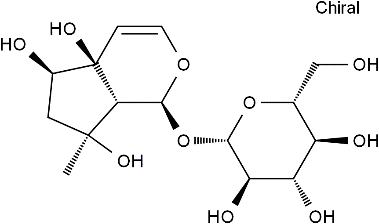 | ↑GRP78 (HSPA5) | IL-1β, IL-6, COX-2, MMP-13, COL2A1, ACAN, Bcl-2, CDK1, Cyclin D1, PFKP | ER stress/AMPK, Glycolysis, NF-κB | In vitro (rat chondrocytes), In vivo (ACLT rat OA model) | Inhibits ER stress via GRP78/p-IRE1α; activates AMPK; suppresses inflammatory cytokines and ECM-degrading enzymes;restores cartilage matrix; improves OA histology. | [221] |
